# Supplementary material for: High precision FRET studies reveal reversible transitions in nucleosomes between microseconds and minutes
Source: Nat Commun. 2018 Nov 6;9:4628. doi: 10.1038/s41467-018-06758-1 (PMC6219519; doi:10.1038/s41467-018-06758-1)
Supplement: Supplementary file 1 — Supplementary Information [file 41467_2018_6758_MOESM1_ESM.pdf]

High precision FRET studies reveal reversible transitions in nucleosomes between microseconds and minutes

Gansen et al.

Information	Content	Page
Supplementary Figure 1	Single molecule sub-ensemble (se) fluorescence decay analysis of 20 pM $I_\beta I_\alpha$-wt nucleosomes at 150 mM NaCl	**2**
Supplementary Figure 2	Mean interdye distances $\langle R_{DA}{}^{LF}\rangle_E$ and $\langle R_{DA}{}^{MF}\rangle_E$ for $I_\beta I_\alpha$-wt nucleosomes	**3**
Supplementary Figure 3	Dissociation kinetics for 20 pM $I_\beta I_\alpha$-wt nucleosomes at 1150, 1000, 850 and 500 mM NaCl.	**5**
Supplementary Figure 4	Quality of reconstitution for wild type ("$I_\beta I_\alpha$ -wt") and H2A-R81A ("$I_\beta I_\alpha$ -mut") nucleosomes visualized on a native 6% polyacrylamide gel (60:1 acrylamide:bisacrylamide).	**6**
Supplementary Figure 5	Filtered FCS in steps	**8**
Supplementary Table 1	Relationships between average distances ⟨RDA⟩E, ⟨RDA⟩ and Rmp.	**10**
Supplementary Table 2	seTCSPC analysis results for 20 pM $I_\beta I_\alpha$-wt nucleosomes at 150 mM NaCl.	**10**
Supplementary Table 3	Differently weighted average fluorescence lifetimes conversion.	**11**
Supplementary Table 4	Fluorophore anisotropies in the MF/MF* subspecies of $I_\beta I_\alpha$-wt nucleosomes at 150 mM NaCl.	**11**
Supplementary Table 5	Midpoint concentration and the half-width of the transition b.	**11**
Supplementary Table 6	Summarized characteristic time scales from Supplementary Fig. 3a-d.	**12**
Supplementary Note 1	Time-resolved fluorescence decay analysis	**13**
Supplementary Note 2	The kinetic model of nucleosome disassembly	**15**
Supplementary Note 3	Parameterization of the geometric model for $I_\beta I_\alpha$-nucleosomes	**22**
Supplementary Note 4	Species-selective filtered FCS	**27**
Supplementary Note 5	Characterization of nucleosome disassembly by salt-induced destabilization using ensemble FRET measurements	**28**
Supplementary Note 6	Analysis of the donor and acceptor mobility in MF/MF* via fluorescence anisotropy	**29**
Supplementary Methods	• DNA sequence and nucleosome labeling<br>• Analysis of multi-parameter smFRET data	**30**
Supplementary References		**32**

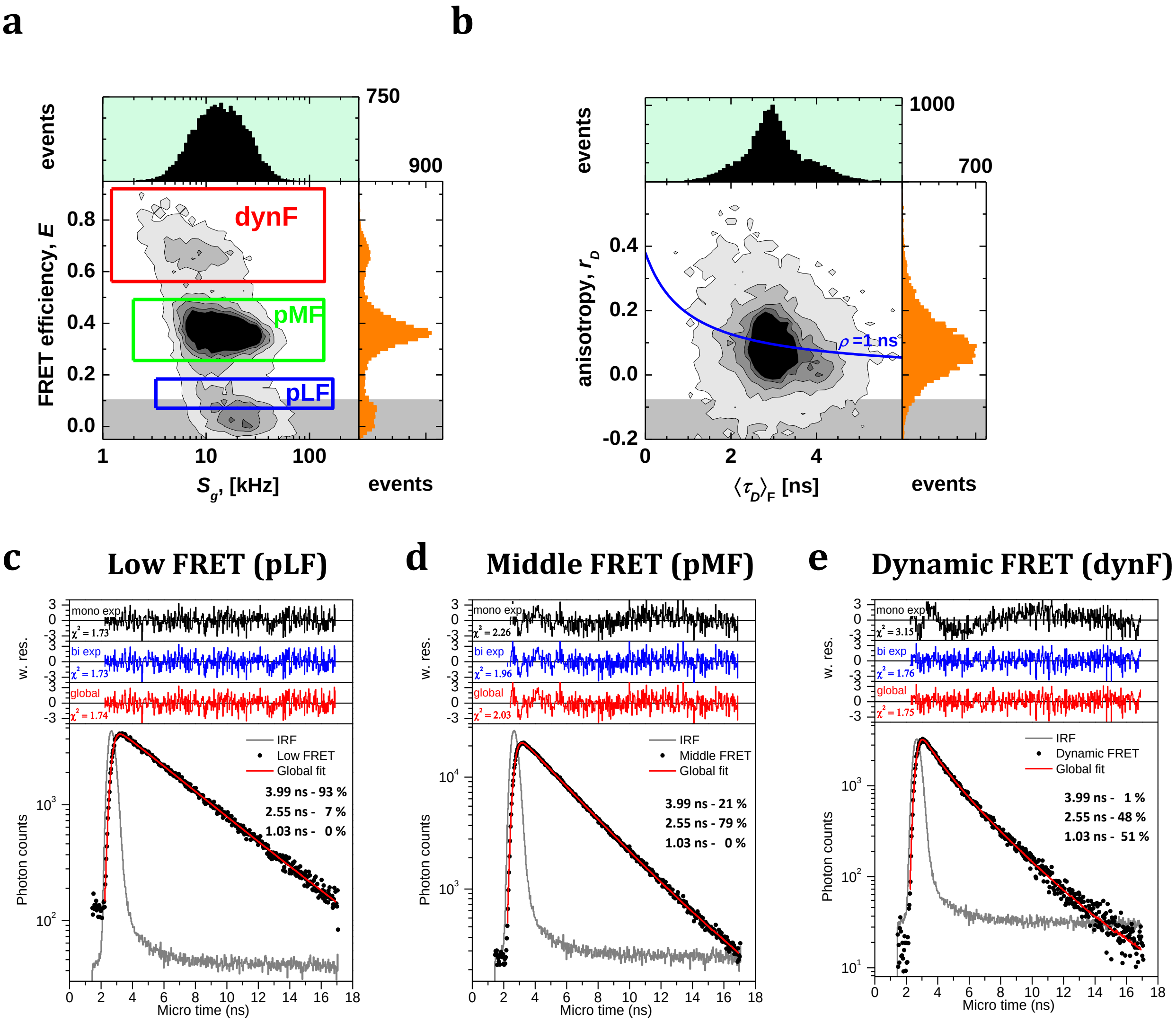

Supplementary Fig. 1. **Single molecule sub-ensemble (se) fluorescence decay analysis of 20 pM $I_\beta I_\alpha$-wt nucleosomes at 150 mM NaCl. a)** Selection of pLF, pMF and dynF population for sub-ensemble lifetime analysis. S_g denotes the burst-wise count rate in the green channel. **b)** 2D histogram of fluorescence lifetime $\langle\tau_D\rangle_F$ distributions on x axis and scatter corrected anisotropy r_D on y axis. The overlaid blue curve shows the Perrin equation with a fundamental anisotropy r_0 = 0.38 and the species-specific rotational correlation time ρ = 1 ns. **c-e)** Fluorescence lifetime decay histograms from pLF, pMF and dynF selections (black dots). Data were approximated by three different models; the respective residuals are compared in the top panels: individual mono-exponential fits (black), individual bi-exponential fits (blue) and a global fit of all decays with three lifetimes (red). Fit results are summarized in Supplementary Tables 1-2. Residuals for a fit with donor-only and 1 FRET species with linker diffusion are shown in the **middle panel**. Data were significantly better described by two FRET components.

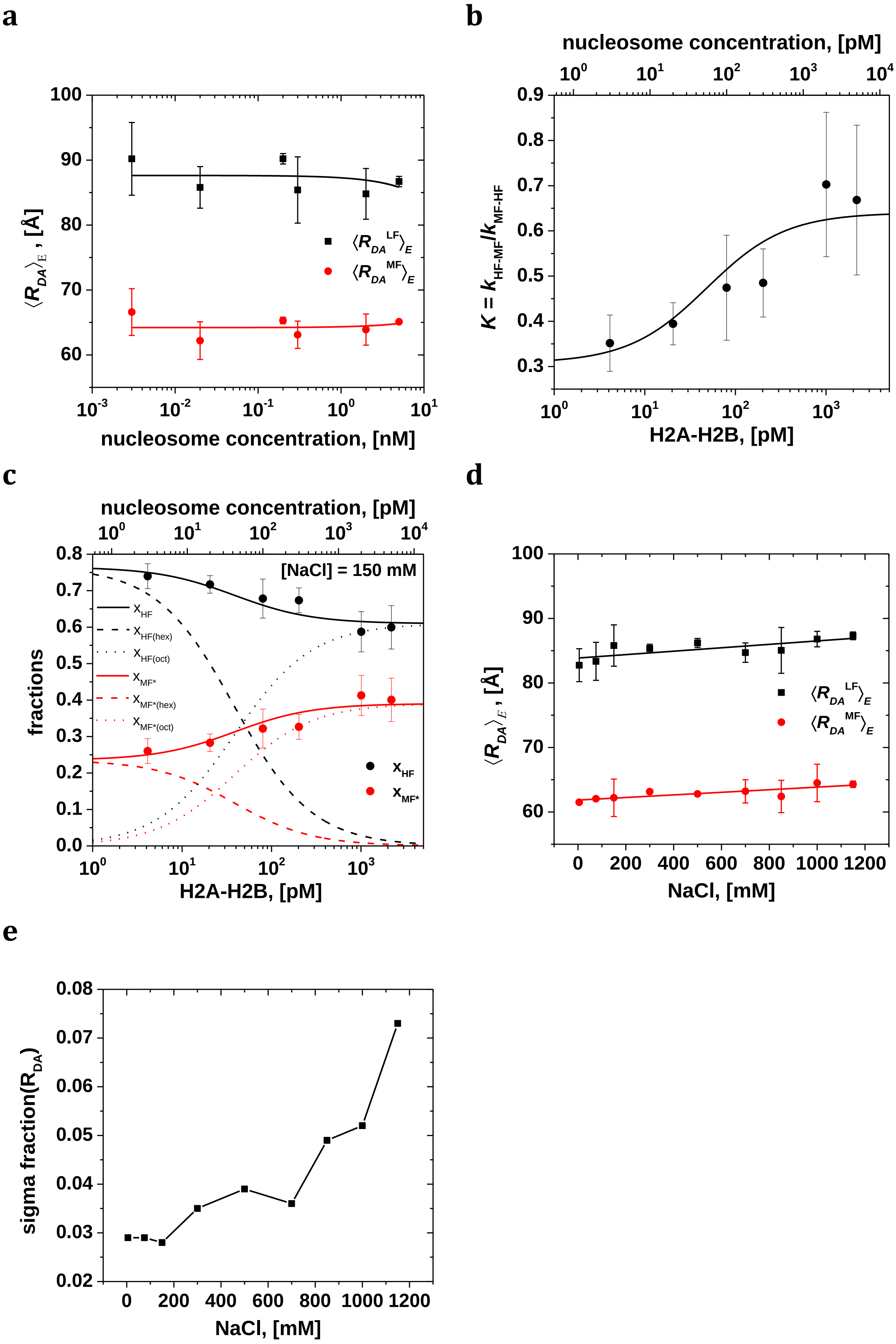
a
b
c
d
e
nucleosome concentration, [nM]
nucleosome concentration, [pM]
H2A-H2B, [pM]
NaCl, [mM]
fractions
sigma fraction(R_DA)
K = k_HF-MF/k_MF-HF
[NaCl] = 150 mM
x_HF
x_MF*
x_HF(hex)
x_HF(oct)
x_MF*(hex)
x_MF*(oct)
⟨R_DA^LF⟩_E
⟨R_DA^MF⟩_E
⟨R_DA⟩_E , [Å]

Supplementary Fig. 2. Mean interdye distances $\langle R_{DA}{}^{\mathrm{LF}}\rangle_{\mathrm{E}}$ and $\langle R_{DA}{}^{\mathrm{MF}}\rangle_{\mathrm{E}}$ for $\mathrm{I}_{\beta}\mathrm{I}_{\alpha}$-wt nucleosomes. a) Dependence on the nucleosome concentration at 150 mM NaCl with linear regression of the data. Resulting fit equations are: $\langle R_{DA}{}^{\mathrm{LF}}\rangle_{\mathrm{E}}$ /[Å] = (87.6 ± 1.3) - (0.4 ± 0.6)·c_{nuc}/[nM] and $\langle R_{DA}{}^{\mathrm{MF}}\rangle_{\mathrm{E}}$ /[Å] = (64.2 ± 0.9) + (0.1 ± 0.4)·c_{nuc}/[nM], indicating no significant concentration dependence of the mean distances $\langle R_{DA}\rangle_E$. **b, c)**: Dependence of the equilibrium constant for HF-MF* interconversion (**b**) and species fractions (**c**) in dynF population on the H2A-H2B concentration. Data points are from dynPDA analysis of single molecule experiments with error bars being standard errors of the mean from different measurements. Lines are simulated using fit parameters obtained from the kinetic model Supplementary Equations 6-15 (see Fig. 4f). **d)** Mean interdye distances $\langle R_{DA}{}^{\mathrm{MF}}\rangle$ and $\langle R_{DA}{}^{\mathrm{LF}}\rangle$ for $\mathrm{I}_{\beta}\mathrm{I}_{\alpha}$-wt nucleosomes as a function of NaCl concentration at 20 pM nucleosomes (compare other fit data by dynPDA Fig. 5) with linear regression of the data. Resulting fit equations are: $\langle R_{DA}{}^{\mathrm{LF}}\rangle_{\mathrm{E}}$ /[Å] = (83.9 ± 0.6) + (0.003 ± 0.001)·c_{NaCl}/[mM] and $\langle R_{DA}{}^{\mathrm{MF}}\rangle_{\mathrm{E}}$ /[Å] = (61.8 ± 0.3) + (0.0020 ± 0.0005)·c_{NaCl}/[mM]. A small but significant increase of both distances with increasing salt concentration is observed. **e)** Width sigma$\left(R_{DA}^{(i)}\right)$ of i-th static Gaussian was assumed proportional to the mean donor-acceptor distance, $\langle R_{DA}^{(i)}\rangle$[1] sigma$\left(R_{DA}^{(i)}\right) = \mathrm{sigma}_{\mathrm{fraction}(R_{DA})} \cdot \langle R_{DA}^{(i)}\rangle$, and one $\mathrm{sigma}_{\mathrm{fraction}(R_{DA})}$ value was fitted for all static distributions at each salt concentration. With increasing NaCl concentration $\mathrm{sigma}_{\mathrm{fraction}(R_{DA})}$ becomes larger indicating on looser static structures at high salt conditions. Error bars are standard errors from at least three different measurements.

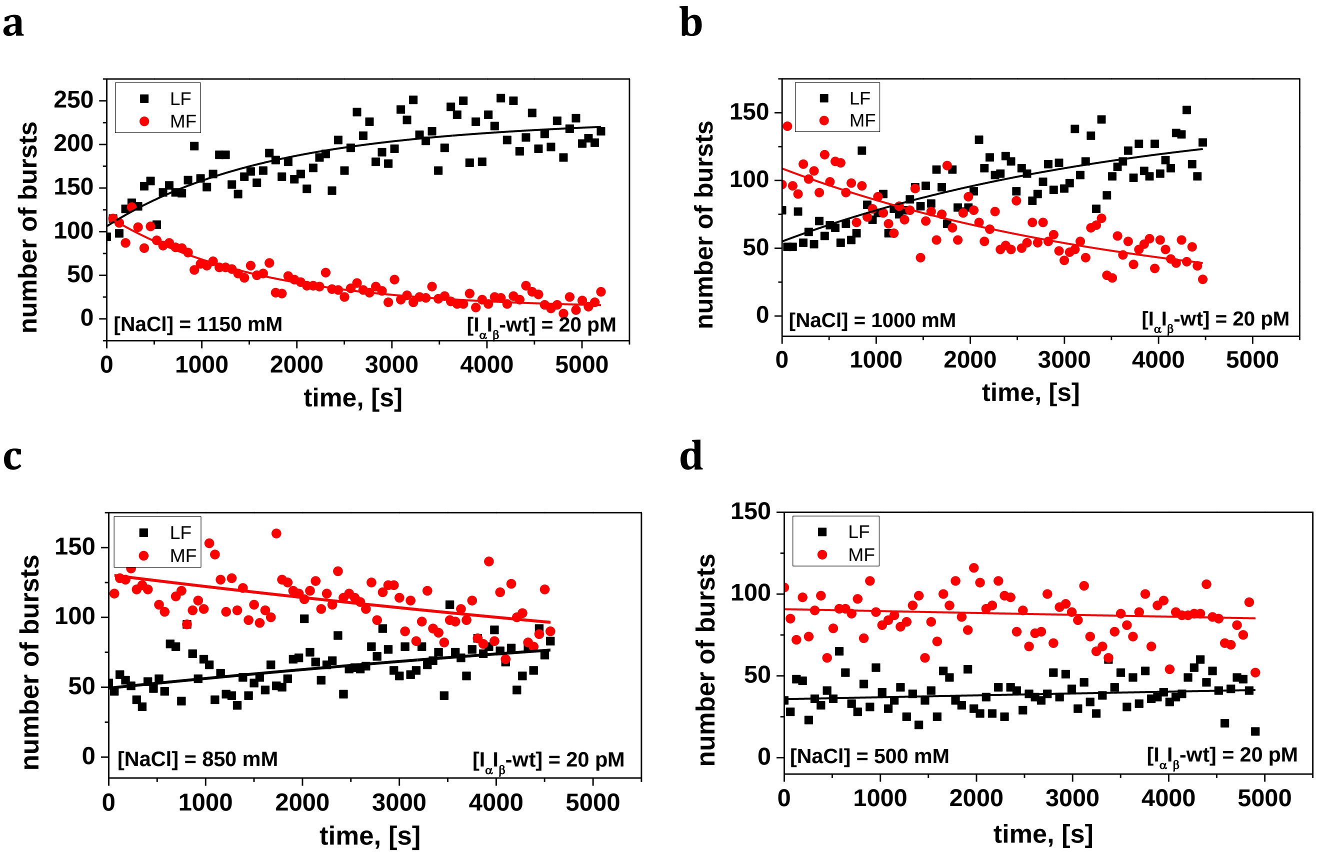

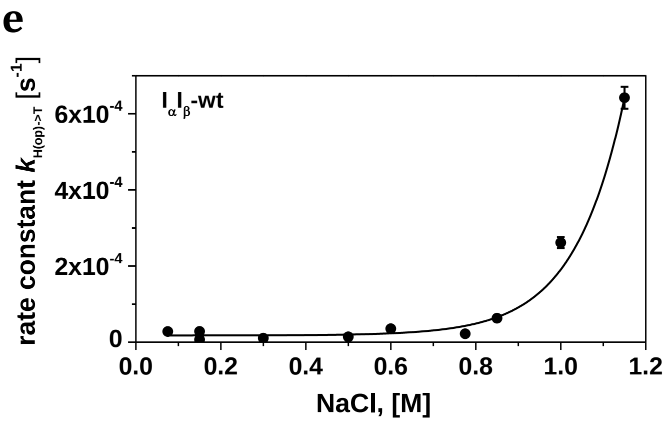

$$k_{H(op)\to T} = k^{0}_{H(op)\to T} + k^{0'}_{H(op)\to T} \cdot e^{[\mathrm{NaCl}]/c}\ \mathrm{s}^{-1}$$

$$k^{0}_{H(op)\to T} = (1.8 \pm 0.5)\times 10^{-5}\ \mathrm{s}^{-1}$$
$$k^{0'}_{H(op)\to T} = (3.4 \pm 4.9)\times 10^{-8}\ \mathrm{s}^{-1}$$
$$c = (117 \pm 17)\ \mathrm{mM}$$

Supplementary Fig. 3. Salt dependence of disassembly of I$_\beta$I$_\alpha$-wt nucleosomes. a-e) Dissociation kinetics for 20 pM I$_\beta$I$_\alpha$-wt nucleosomes at 1150, 1000, 850 and 500 mM NaCl. All data were analyzed as described in Supplementary Note 2, step V). Fit parameters are given with standard errors from the fit.

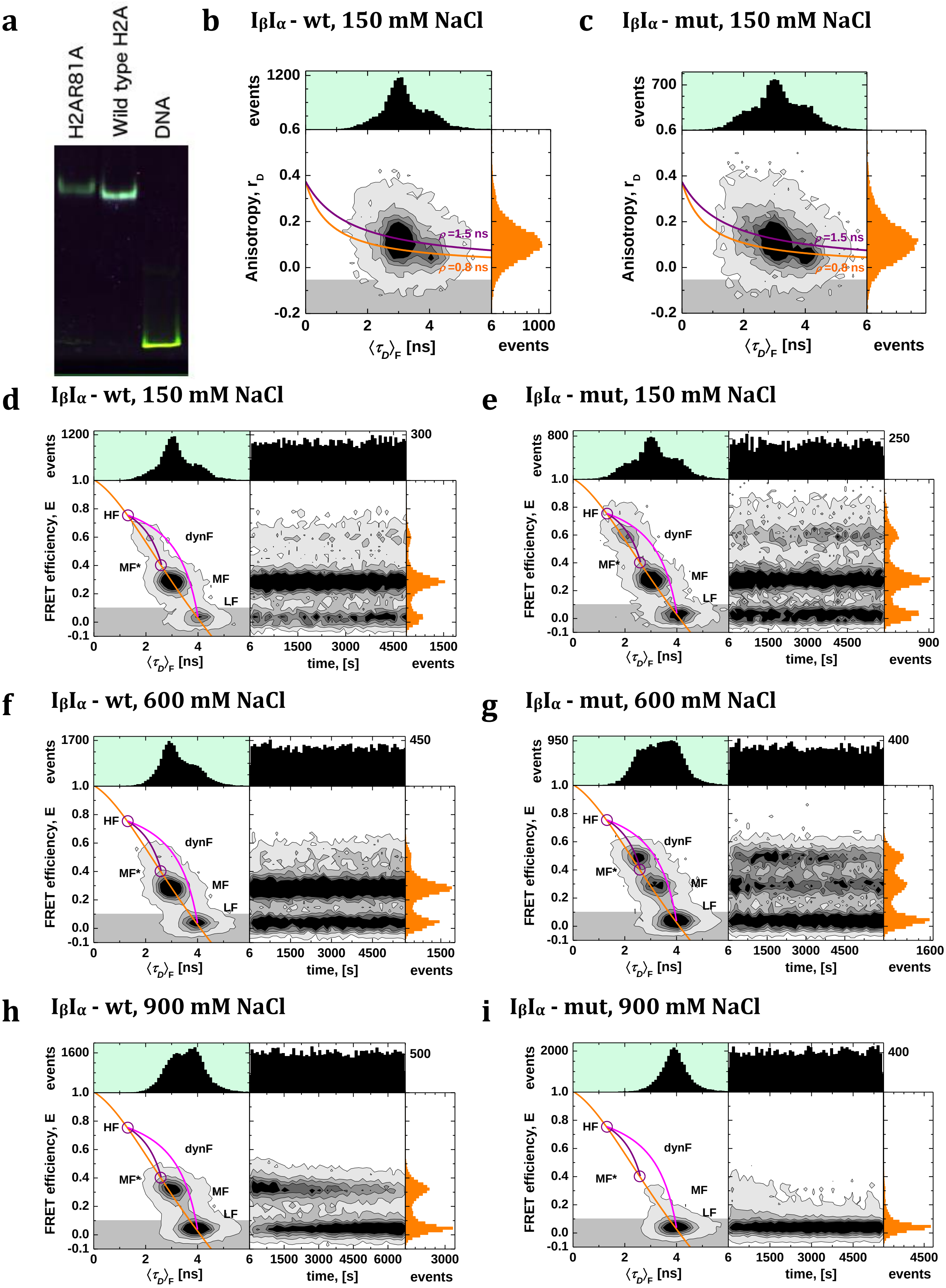
a
H2AR81A
Wild type H2A
DNA
b
IβIα - wt, 150 mM NaCl
c
IβIα - mut, 150 mM NaCl
Anisotropy, r_D
ρ =1.5 ns
ρ =0.8 ns
⟨τ_D⟩_F [ns]
events
d
IβIα - wt, 150 mM NaCl
e
IβIα - mut, 150 mM NaCl
f
IβIα - wt, 600 mM NaCl
g
IβIα - mut, 600 mM NaCl
h
IβIα - wt, 900 mM NaCl
i
IβIα - mut, 900 mM NaCl
FRET efficiency, E
HF
dynF
MF*
MF
LF
time, [s]

j

k

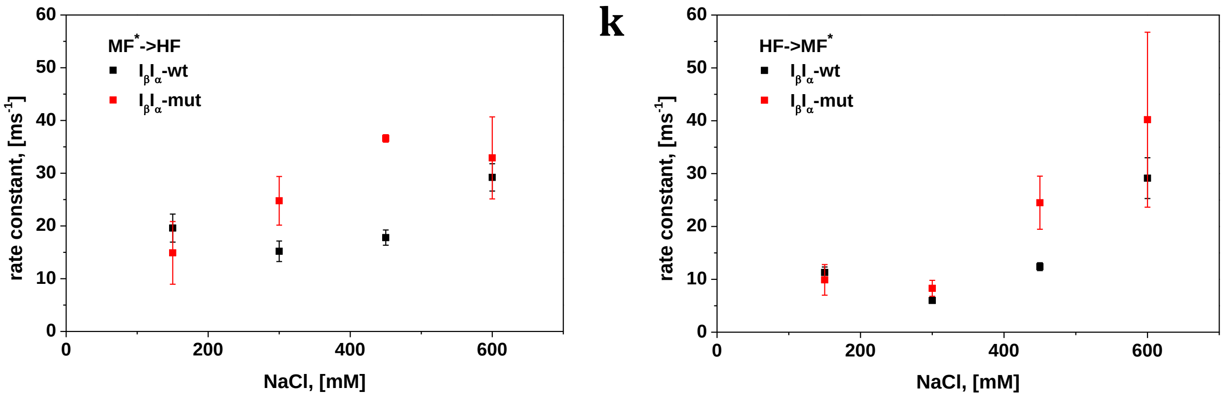

Supplementary Fig. 4. Properties of wild type (“$I_\beta I_\alpha$-wt”) and H2A-R81A (“$I_\beta I_\alpha$-mut”). a) Quality of reconstitution for wild type (“$I_\beta I_\alpha$-wt”) and H2A-R81A (“$I_\beta I_\alpha$-mut”) nucleosomes visualized on a native 6% polyacrylamide gel (60:1 acrylamide:bisacrylamide). Electric field strength was set to 10 V/cm. The running buffer was TBE adjusted to pH 7.5. **b-i)** The effect of the H2A-R81A mutation on nucleosome dynamics was tested in MFD experiments. All nucleosome samples were diluted to 100 pM (20 pM labeled and 80 pM unlabeled nucleosomes). The donor anisotropy, r_D, vs average fluorescence lifetime data are shown for **b)** $I_\beta I_\alpha$-wt nucleosomes at 150 mM NaCl, **c)** $I_\beta I_\alpha$-mut. nucleosomes at 150 mM NaCl. Insertion of R81A in

H2B does not affect local fluorophore dynamics. MFD data are shown for **d)** $I_\beta I_\alpha$-wt nucleosomes at 150 mM NaCl, **e)** $I_\beta I_\alpha$-mut nucleosomes at 150 mM NaCl, **f)** $I_\beta I_\alpha$-wt nucleosomes at 600 mM NaCl and **g)** $I_\beta I_\alpha$-mut nucleosomes at 600 mM NaCl. At 600 mM H2A-R81A considerably destabilized nucleosomes by first promoting rapid accumulation of dynF followed by an increase in LF subspecies. **h)** $I_\beta I_\alpha$-wt nucleosomes at 900 mM NaCl and **i)** $I_\beta I_\alpha$-mut nucleosomes at 900 mM NaCl. At 900 mM $I_\beta I_\alpha$-wt nucleosomes are considerably destabilized and via dynF an increase in LF subspecies is visible. At 900 mM $I_\beta I_\alpha$-mut nucleosomes are fully destabilized and of the majority of detected bursts is assigned to the LF subspecies. **j, k)** Kinetic rate constants for the MF*⇄HF transition as a function of NaCl concentration. Error bars are standard errors from at least three different measurements.

Selections of LF and HF TWs for filter generation.

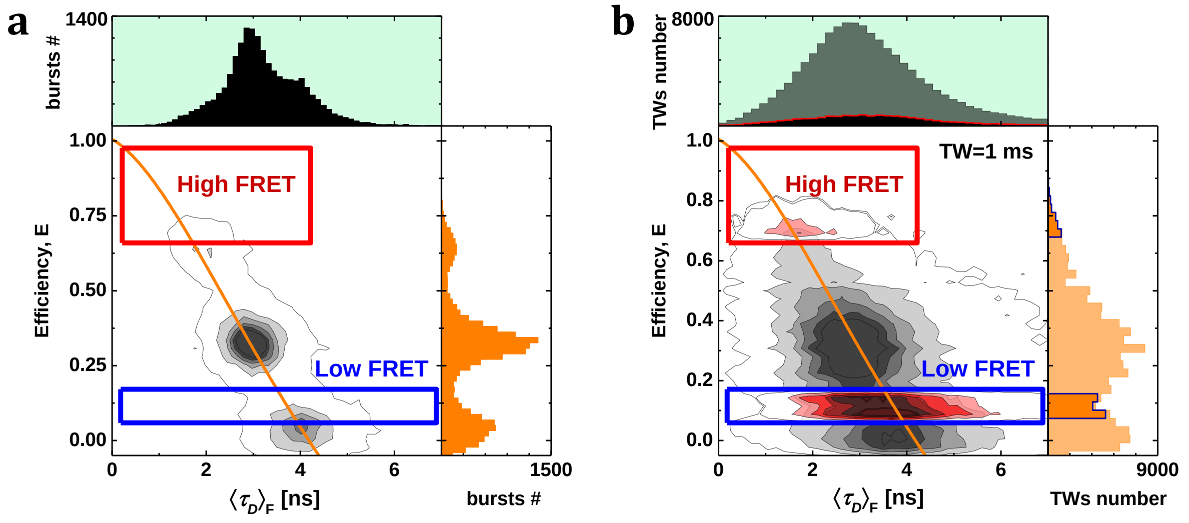

Corresponding fluorescence decay histograms

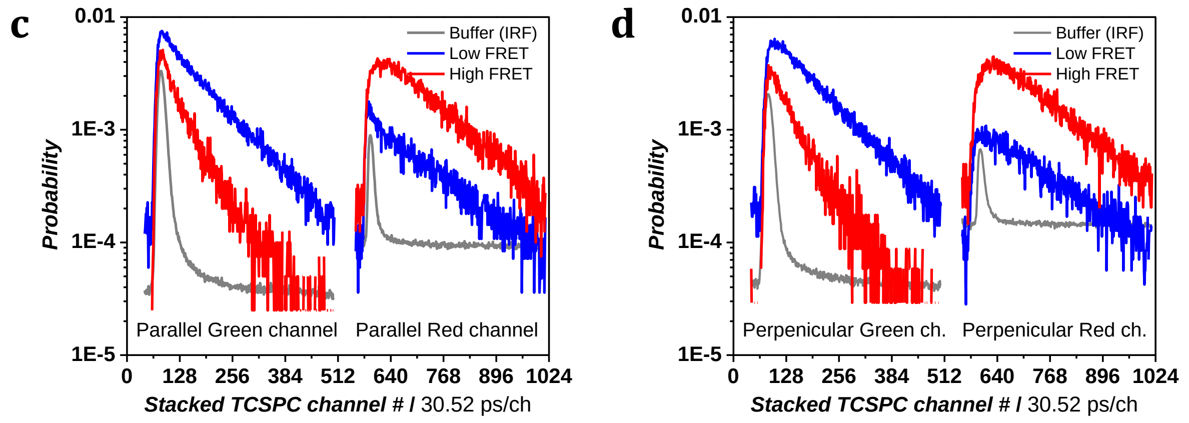

Generated filters

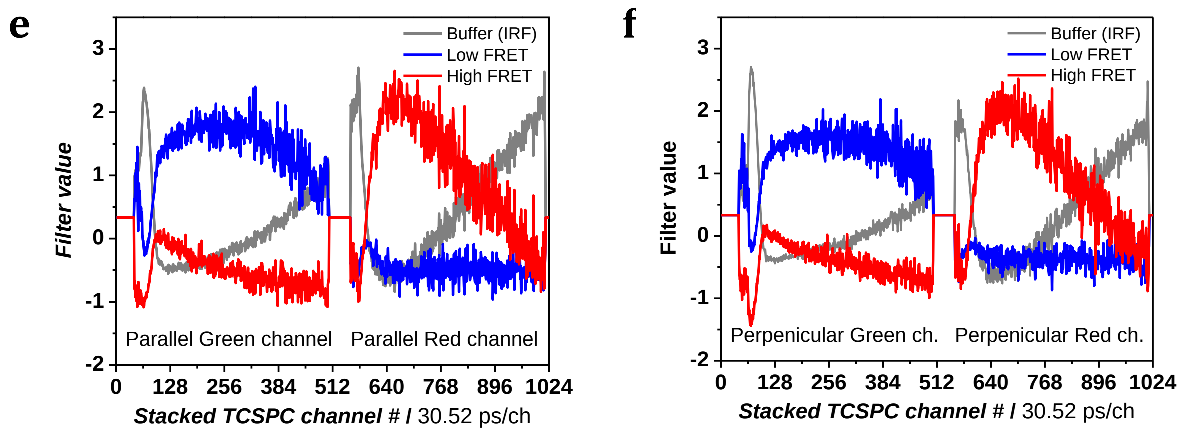

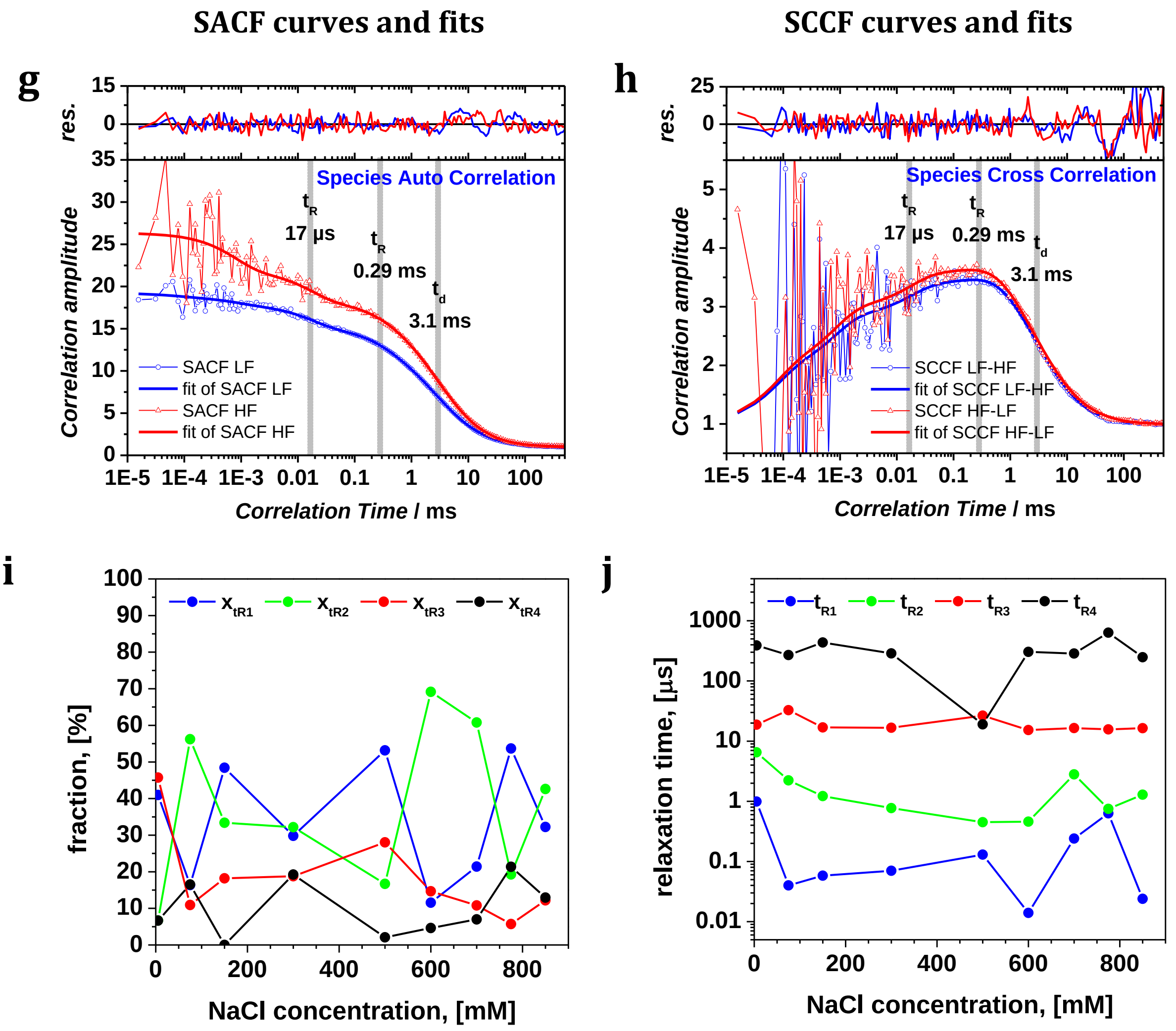

Supplementary Fig. 5. Filtered FCS of $I_\beta I_\alpha$ nucleosome dynamics in steps: **a)** MFD 2D plot: FRET efficiency, E vs fluorescence weighted averaged lifetime, $\langle \tau_D \rangle_F$ for burstwise analysis of $I_\beta I_\alpha$-wt nucleosomes at 300 mM NaCl, and **b)** corresponding MFD 2D plot for TW=1 ms analysis. High FRET (red box) and low FRET (blue box) species are indicated on both plots. As one can notice there are more events (TW) in the red box (HF) in the time windows analysis in plot B. Selected TWs are also indicated by red overlays in the 2D plot and the corresponding 1D projections on both axes. Corresponding stacked fluorescence decay histograms from **c)** parallel green and parallel red and **d)** perpendicular green and perpendicular red detection channels for buffer (gray), Low FRET (LF) and High FRET (HF) selected TWs are presented. Based on these decay patterns 3 component filters for **e)** parallel and **f)** perpendicular signals were generated as described in [2]. **g)** Species Auto Correlation Functions (SACF) and **h)** Species Cross Correlation Functions (SCCF) for LF (blue symbols) and HF (red symbols) species were generated using these filters. Generated curves were fitted globally using four global relaxation times in bunching (SACF) and anticorrelation (SCCF) terms in addition to a 3D Gaussian diffusion term with global diffusion time, t_d. Fit curves are overlaid as continuous lines with corresponding colors. Fitted fractions of anticorrelation terms in **i)** SCCF and **j)** global relaxation times as function of salt concentration, [NaCl] are presented.

Supplementary Table 1: Relationships between average distances $\langle R_{DA}\rangle_E$, $\langle R_{DA}\rangle$ and R_{mp}.

For convenience we convert average distances from different data-sources, i.e. ($\langle R_{DA}\rangle$ - from TCSPC, $\langle R_{DA}\rangle_E$ - from PDA, and R_{mp} - from coarse-grained structural modeling). To generate conversion functions we simulate typical accessible volumes (AVs) for proteins and nucleic acids [3,4] . These AVs are systematically translated / rotated and the average distances $\langle R_{DA}\rangle_E$, $\langle R_{DA}\rangle$, and R_{mp} are calculated. This generates conversion tables which are approximated by 3rd order polynomials, which have an approximation error of 1.7 Å.

	$\langle R_{DA}\rangle$ from R_{mp}	$\langle R_{DA}\rangle_E$ from R_{mp}	$\langle R_{DA}\rangle_E$ from $\langle R_{DA}\rangle$	$\langle R_{DA}\rangle$ from $\langle R_{DA}\rangle_E$
C_0	8.2377	20.498	15.323	-25.586
C_1	0.8026	0.4778	0.59283	1.799
C_2	0.0019858	0.0030095	0.0017537	-0.0063039
C_3	-0.000007026356	-0.0000016467	0.0000032013	0.000013171

Supplementary Table 2: seTCSPC analysis results.

Comparison of sub-ensemble lifetime fits for 20 pM $I_\beta I_\alpha$-wt nucleosomes at 150 mM NaCl (data are shown in Supplementary Fig. 1) using a series of exponentials.

Selected population	Formal bi-exponential fit (Supplementary Equation 1, N=2): individual lifetimes $\langle\tau_D\rangle_x{}^i$ and fractions		Joint fit (Supplementary Equation 1, N=3) with three global lifetimes $\langle\tau_D\rangle_x{}^i$		
	$\langle\tau_D\rangle_x{}^1$	$\langle\tau_D\rangle_x{}^2$ constant offset needed – 1.9 counts per TAC bin	$\langle\tau_D\rangle_x{}^{HF}$ =1.03 ns	$\langle\tau_D\rangle_x{}^{MF*}$ =2.55 ns	$\langle\tau_D\rangle_x{}^{LF}$ =3.99 ns
			**and individual fractions**		
LF	3.97 ns (99.7%)	16.5 ns (0.3%)	0 %	7 %	93 %
MF	2.71 ns (88 %)	4.67 ns (12 %)	0 %	79 %	21 %
dynF	1.05 ns (52 %)	2.61 ns (48 %)	51 %	48 %	1 %
**equivalent distances**					
Mean distances $\langle R^i_{DA}\rangle$ computed from the global $\langle\tau_D\rangle_x{}^i$ by eq. *a*.	-	-	45.2 Å	60.4 Å	101.2 Å
$\langle R_{DA}\rangle_E$			46.0 Å	57.2 Å	96.6 Å

eq. *a*: $$\langle R_{DA}{}^i\rangle = R_0 \cdot \left(\frac{\langle\tau_D\rangle_x{}^i}{\tau_{D0} - \langle\tau_D\rangle_x{}^i}\right)^{\frac{1}{6}}, \quad where\ R_0 = 55.6\text{Å}, \quad \tau_{D0} = 4.1ns$$

Supplementary Table 3: Differently weighted average fluorescence lifetimes conversion.

Conversion of species-weighted average lifetimes ($\langle\tau_D\rangle_x$ - fit results in **Supplementary Table 2**) into fluorescence-weighted average lifetimes ($\langle\tau_D\rangle_F$) by 4th order polynomial with coefficients C_0 = -0.0211, C_1 = 0.434, C_2 = 0.3719, C_3 = -0.0798 and C_4 = 0.0056 obtained by numerical simulation (see in <u>Computation of the theoretical static FRET line corrected for dye linker dynamics</u>):, where values for width of the linker broadening $\sigma_{R_{DA}} = 6\,\text{Å}$, $\tau_{D(0)}$ = 4.1 ns, $\Phi_{FD(0)}$ =0.8 and effective Φ_{FA} =0.32 were used.

Simulated data (black circles) and polynomial fit (red line) are presented below.

Species-weighted		Fluorescence-weighted	
$\langle\tau_D\rangle_x^{LF}$	3.97 ns	$\langle\tau_D\rangle_F^{LF}$	4.00 ns
$\langle\tau_D\rangle_x^{MF}$	2.71 ns	$\langle\tau_D\rangle_F^{MF}$	2.80 ns
$\langle\tau_D\rangle_x^{MF*}$	2.55 ns	$\langle\tau_D\rangle_F^{MF*}$	2.65 ns
$\langle\tau_D\rangle_x^{HF}$	1.03 ns	$\langle\tau_D\rangle_F^{HF}$	1.30 ns

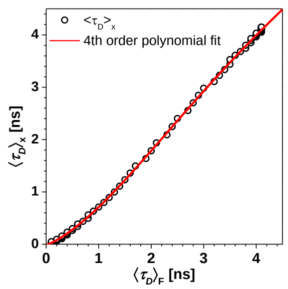

Supplementary Table 4: Fluorophore anisotropies.

Fluorophore anisotropies of the MF/MF* subspecies for $I_\beta I_\alpha$-wt nucleosomes at 150 mM NaCl (discussion see Supplementary Note 6).

[nucleosomes]	Donor anisotropies		Acceptor anisotropies	
	r_1	r_2	r_1	r_2
20 pM	0.114		0.086 (18%)	0.017 (82%)
100 pM	0.100 (85%)	0.195 (15%)		0.054
300 pM	0.119		0.089 (10%)	0.041 (90%)
2 nM	0.107		0.12 (47%)	0.038 (53%)
5 nM	0.109 (90%)	0.198 (10%)	0.085 (54%)	0.018 (46%)

Supplementary Table 5: Midpoint concentration $c_{1/2}$ and the half-width of the transition *b*.

Characterization ($c_{1/2}$ and *b*) of nucleosome disassembly by salt-induced destabilization using ensemble FRET studies in a plate reader (for details see Supplementary Note 5, fitted by Supplementary Equation 36).

FRET pair and (species)	Fig.	$c_{1/2}$ mM	err($c_{1/2}$) mM	b mM	err(b) mM	dP/dc 1/M	err(dP/dc) 1/M	err(dP/dc) %
H2B-Dy_α(D_αA)	2c	546	39	116.63	28.12	-2.1	0.5	24
$E_\alpha Dy_\beta$	3	631	6	105.02	5.75	-2.4	0.1	5
$E_\beta Dy_\alpha$	3	694	12	168.10	12.67	-1.5	0.1	8
$I_\beta I_\alpha$	6b	783	5	30.29	4.80	-8.3	1.3	16
H2B-Dy_α($D_\alpha D_\beta$A)	2c	813	10	26.98	10.50	-9.3	3.6	39
H2B-Dy_α(D_βA)	2c	866	33	148.30	29.59	-1.7	0.3	20

Supplementary Table 6: Summarized characteristic time scales from Supplementary Fig. 3a-d.

Dissociation times for 20 pM $I_\beta I_\alpha$-wt nucleosomes at elevated NaCl concentration. Subpopulations are defined as described in Supplementary Fig. 1.

[NaCl]	$k_{H(op)\rightarrow T}$ [s^{-1}]	A	N_{tot}(t=0)	k_{tot} [s^{-1}]
1150 mM	(6.4±0.3)x10^{-4}	113±5	224±5	(1.0±0.8)x10^{-5}
1000 mM	(2.6±0.2)x10^{-4}	106±3	164±4	(-0.2±1.0)x10^{-5}
850 mM	(6.3±0.8)x10^{-5}	128±3	180±5	(-0.8±1.0)x10^{-5}
500 mM	(1.4±0.8)x10^{-5}	89±2	127±4	(-0.6±1.1)x10^{-5}

Supplementary Note 1: Time-resolved fluorescence decay analysis

Model functions

The fluorescence decay of the donor in the absence of acceptor can be multi-exponential due to local quenching. To account for these effects the donor only reference samples were fitted by a multi-exponential relaxation model.

$$F_{\mathrm{D(0)}}(t) = \sum_i x_{\mathrm{D(0)}}^{(i)} \cdot \exp(-t / \tau_{\mathrm{D(0)}}^{(i)}) \qquad \text{(Supplementary Equation 1)}$$

Here $\tau_{\mathrm{D(0)}}^{(i)}$ are the donor fluorescence lifetime components and $x_{\mathrm{D(0)}}^{(i)}$ the pre-exponential factors.

Multi-exponential donor decays were accounted for in the analysis of the FRET samples by global fitting. We assumed that all donor species are quenched by the same FRET rate constant k_{RET}. This is true if quenching does not change the donor radiative lifetime and when FRET is uncorrelated with the donor quenching. Based on these assumptions, the donor fluorescence intensity decay in the presence of acceptor dye $F_{D(A)}(t)$ can be factorized into the donor fluorescence decay in absence of FRET and the time-resolved FRET-induced donor quenching $\varepsilon_{D(A)}(t)$:

$$F_{D(A)}(t) = F_{D(0)}(t) \cdot \varepsilon_{D(A)}(t) \qquad \text{(Supplementary Equation 2)}$$

We relate the FRET-induced donor decay to the distribution of distances by the rate-constant of energy transfer as defined by Förster:

$$k_{RET} = k_F \cdot \kappa^2 \cdot \left(\frac{R_{0J}}{R_{DA}} \right)^6$$

Here, R_{0J} is a reduced Förster-radius, k_F - the radiative rate constant of fluorescence and κ^2 is the orientation-factor. The reduced Förster-radius is given by:

$$R_{0J} = \left[\frac{9(\ln 10)}{128\pi^5 \cdot N_A} \cdot \frac{J}{n^4} \cdot \right]^{\frac{1}{6}} = 0.2108 \cdot \text{Å} \cdot \left[\frac{1}{n^4} \cdot \left(\frac{J(\lambda)}{\mathrm{mol}^{-1} \cdot \mathrm{dm}^3 \cdot \mathrm{cm}^{-1} \cdot \mathrm{nm}^4} \right) \right]^{\frac{1}{6}}$$

where N_A is Avogadro's constant, n is the refractive index of the medium and $J = \int f_D(\lambda) \cdot \varepsilon_A(\lambda) \cdot \lambda^4 \cdot d\lambda$ is the overlap integral between $f_D(\lambda)$, the donor emission spectrum and $\varepsilon_A(\lambda)$, the acceptor absorption spectrum. This reduced Förster-radius stresses that the FRET-rate constant is independent of quenching and specific for the dye-pair under the condition that the spectral overlap is independent of dynamic quenching. With these assumptions, the FRET-induced donor decay relates to the distance distribution $p(R_{DA})$ by:

$$\varepsilon_{D(A)}(t) = \int p(R_{DA}) \cdot \exp\left(-t \cdot \langle \kappa^2 \rangle \cdot k_{\mathrm{F}} \cdot \left[1 + (R_{0\mathrm{J}} / R_{\mathrm{DA}})^6\right]\right) dR_{DA} \qquad \text{(Supplementary Equation 3)}$$

Usually the orientation factor can be approximated by an average $\langle\kappa^2\rangle \approx 2/3$. We used a reduced Förster-radius of R_{0J}=55.6 Å which was determined for the donor Alexa488 with a radiative rate constant k_F=0.2239 ns^{-1}.

In the fit we used continuous distance distributions which are described by a superposition of normal distributions:

$$p(R_{DA}) = \sum_{i=1}^{N} x_{DA}^{(i)} \cdot \frac{1}{w_{\mathrm{DA}} \sqrt{\pi / 2}} \cdot \exp\left(-2 \cdot \left[\frac{R_{\mathrm{DA}} - \langle R_{DA}^{(i)} \rangle}{w_{DA}} \right]^2 \right) \qquad \text{(Supplementary Equation 4)}$$

Here, $\left\langle R_{DA}^{(i)} \right\rangle$ is the mean of the state (i) distance distribution with species fraction $x_{DA}^{(i)}$ and a width w_{DA} set to a physical meaningful value of 12 Å (flexible dye-linkers) estimated from dye clouds AV simulations.

The final analysis model is obtained by substituting Supplementary Equation 4 into Supplementary Equation 3 and Supplementary Equation 3 into Supplementary Equation 2. Finally, the fluorescence intensity decay of the donor in presence and absence of FRET (Supplementary Equation 5) are globally fitted. By the global (joint) analysis of the reference sample and the FRET-sample the photo-physical properties (dynamic quenching) of the donor dye are taken into account.

Sub-ensembleTCSPC

The selected model function was fit to the experimental fluorescence intensity decays using the iterative re-convolution approach. Here, the model-decay curves were convoluted with the experimental instrument response function (*IRF*). Furthermore a constant offset c of the fluorescence intensity was considered. Finally, to correct for the instrumental differential non-linearity the response to uncorrelated light was recorded and considered in the fitting procedure by multiplying the model-function with the normalized/smoothed uncorrelated instrumental response *Lin*. Given these corrections the experimental time-resolved fluorescence intensities of the FRET-sample and the donor reference sample are presented as:

$$
\begin{aligned}
F_{\mathrm{FRET}}(t) &= \left(N_0 \cdot \left[(1 - x_{\mathrm{DOnly}}) \cdot F_{\mathrm{D(A)}}(t) + x_{\mathrm{DOnly}} \cdot F_{\mathrm{D(0)}}(t)\right] \otimes IRF + sc \cdot IRF + c\right) \cdot Lin \\
F_{\mathrm{Ref}}(t) &= \left(N_0 \cdot F_{\mathrm{D(0)}}(t) \otimes IRF + sc \cdot IRF + c\right) \cdot Lin
\end{aligned}
\qquad \text{(Supplementary Equation 5)}
$$

Here, *sc* takes into account scattered light from the sample. The normalized to unit area model functions were scaled by the experimentally measured photon number N_0. This reduces the number of free fitting parameters by 1.

Supplementary Note 2: The kinetic model of nucleosome disassembly

Kinetic scheme (description and justification).

A full kinetic scheme for nucleosome disassembly through sequential loss of H2A-H2B heterodimers was presented in Fig.8, where the two heterodimers are shown in orange and dark yellow, while the tetramer is shown in blue-green. I) Disassembly of the intact nucleosome (octasome, O^{MF}) is predominantly initiated from the weaker binding α-side (see below), where opening of the first dimer:tetramer interface precedes dimer eviction ($O_{cl}^{MF'}$). II-IV) The second dimer can reversibly detach from the tetramer once the first dimer:tetramer interface is broken (O_{op}^{HF}). Once the first dimer is lost ($H_{cl}^{MF'}$), reversible detachment (H_{op}^{HF}) of the remaining dimer proceeds at a faster rate. V) Eventually, after loss of the second heterodimer the remaining tetrasome can adopt variable DNA geometries with most conformations leading to very low or no FRET ($T^{LF/NF}$). At higher salt DNA can fully dissociate from the remaining tetramer (DNA^{NF}).

Consecutive eviction of H2A-H2B dimers leads to two bimolecular (slow) equilibria (steps III and V):

- Internal structural dynamics cause three distinguishable unimolecular (fast) equilibria (steps I, II and IV).
- At least ten rate constants on the way from octasome to tetrasome are involved which are compiled in Table 1.

Besides the FRET analysis in Figs. 2 and 3, the asymmetry in dimer stability at both DNA ends is further supported by species-selective anisotropy analysis of $I_\beta I_\alpha$-wt nucleosomes (**Supplementary Table 4**). After dimer loss, the adjacent DNA is no longer bound to protein, which should increase dye mobility. The fact that donor and acceptor mobility in MF/MF* responded differently to nucleosome concentration suggests that the dimer next to the acceptor (at the α-side) is more prone to eviction. At low nucleosome concentration, the acceptor anisotropy is low while the donor anisotropy is more or less independent.
To solve and find all rate constants we have to make an approximation. Due to different time scales, most steps can be approximated as isolated processes. For convenience, let us start with steps II-IV.

Steps II-IV: The MF* - HF transition

The fast (µs) dynamics between the interconverting species assigned to the *dynF* fraction was found to be dependent on the total nucleosome concentration (Fig. 4f), indicating that a bimolecular step, the eviction/uptake of an H2A-H2B heterodimer, is involved. In the range of concentrations studied ([H2A-H2B] < 10 nM) this bimolecular process, limited by diffusion, takes place on the seconds time scale and cannot be the same that causes the µs dynamics. The simplest model consistent with all observations is the assumption of two fast kinetic systems (similar in their FRET properties), slowly interconverting by a bimolecular process. Thus we postulate the presence of a broken (dynamic) octasome, O_{dyn}, and a hexasome, H, both exhibiting fast interconverting MF* and HF states. Due to the large difference in time scales the equilibria can be treated separately. We can write for the slow process

$$O_{dyn} \rightleftarrows H + \dim, \quad \text{(Supplementary Equation 6)}$$

with O_{dyn} representing the dynamic octasome (Fig. 8, left black box) and H the hexasome (Fig. 8, right black box), each in its two FRET states:

$$[O_{dyn}] = [O_{cl}^{MF*}] + [O_{op}^{HF}] \quad \text{and} \quad [H] = [H_{cl}^{MF*}] + [H_{op}^{HF}]. \quad \text{(Supplementary Equation 7)}$$

The corresponding rate constants are:

$$k(\mathrm{O_{dyn}} \rightarrow \mathrm{H}) = k_{O\rightarrow H} \quad \text{and} \quad k(\mathrm{H} \rightarrow \mathrm{O_{dyn}}) = k_{H\rightarrow O} \cdot [\mathrm{dim}], \qquad \text{(Supplementary Equation 8)}$$

where [dim] denotes the concentration of free H2A-H2B dimer. The mean dissociation constant is defined as

$$K_{dis}^{O/H} = \frac{[\mathrm{H}]\cdot[\mathrm{dim}]}{[\mathrm{O_{dyn}}]} = \frac{k_{O\rightarrow H}}{k_{H\rightarrow O}}. \qquad \text{(Supplementary Equation 9)}$$

With

$$K' = \frac{K_{dis}^{O/H}}{[\mathrm{dim}]} = \frac{[\mathrm{H}]}{[\mathrm{O_{dyn}}]} \qquad \text{(Supplementary Equation 10)}$$

it follows that the fraction of dynamic octasomes is given by

$$x_{oct} = \frac{[\mathrm{O_{cl}^{MF*}}]+[\mathrm{O_{op}^{HF}}]}{[\mathrm{O_{cl}^{MF*}}]+[\mathrm{O_{op}^{HF}}]+[\mathrm{H_{cl}^{MF*}}]+[\mathrm{H_{op}^{HF}}]} = \frac{[\mathrm{O_{dyn}}]}{[\mathrm{O_{dyn}}]+[\mathrm{H}]} = \frac{1}{1+K'} = \frac{1}{1+K_{dis}^{O/H}/[\mathrm{dim}]} \qquad \text{(Supplementary Equation 11)}$$

In the fast equilibrium $\mathrm{MF*} \rightleftarrows \mathrm{HF}$ we describe the observed dependence of the mean exchange rate constants $k_{MF*\rightarrow HF}$ and $k_{HF\rightarrow MF*}$ on nucleosome concentration by assuming that rate constants as obtained by PDA are a weighted sum depending on the fractions of the four involved species in dynamic equilibrium: $\mathrm{O_{cl}^{MF*}}$, $\mathrm{O_{op}^{HF}}$, $\mathrm{H_{cl}^{MF*}}$ and $\mathrm{H_{op}^{HF}}$ with fractions x_{oct}^{MF*}, x_{oct}^{HF}, x_{hex}^{MF*} and x_{hex}^{HF}, respectively. With the definitions: $\sum x_i = 1$, $x^{MF*} = x_{oct}^{MF*} + x_{hex}^{MF*}$, $x^{HF} = x_{oct}^{HF} + x_{hex}^{HF}$, $x_{oct} = x_{oct}^{MF*} + x_{oct}^{HF}$ and $x_{hex} = x_{hex}^{MF*} + x_{hex}^{HF}$ the mean rate constants can be expressed as weighted sum of the corresponding individual rate constants:

$$\begin{aligned} k_{MF*\rightarrow HF} &= \frac{x_{oct}^{MF*}}{x^{MF*}} \cdot k_{O(cl)\rightarrow O(op)} + \frac{x_{hex}^{MF*}}{x^{MF*}} \cdot k_{H(cl)\rightarrow H(op)} \\ &= \left(1+\frac{x_{hex}^{MF*}}{x_{oct}^{MF*}}\right)^{-1} \cdot \left(k_{O(cl)\rightarrow O(op)} - k_{H(cl)\rightarrow H(op)}\right) + k_{H(cl)\rightarrow H(op)} \end{aligned} \qquad \text{(Supplementary Equation 12)}$$

and

$$k_{HF\rightarrow MF*} = \left(1+\frac{x_{hex}^{HF}}{x_{oct}^{HF}}\right)^{-1} \cdot \left(k_{O(op)\rightarrow O(cl)} - k_{H(op)\rightarrow H(cl)}\right) + k_{H(op)\rightarrow H(cl)}. \qquad \text{(Supplementary Equation 13)}$$

In equilibrium we have:

$$\begin{aligned} & x_{oct}^{MF*} \cdot k_{O(cl)\rightarrow O(op)} = x_{oct}^{HF} \cdot k_{O(op)\rightarrow O(cl)} = \left(x_{oct} - x_{oct}^{MF*}\right) \cdot k_{O(op)\rightarrow O(cl)} \\ & \text{or} \quad x_{oct}^{MF*} = x_{oct} \cdot \frac{k_{O(op)\rightarrow O(cl)}}{k_{O(cl)\rightarrow O(op)} + k_{O(op)\rightarrow O(cl)}} \end{aligned} \qquad \text{(Supplementary Equation 14)}$$

and equivalently

$$x_{hex}^{MF*} = x_{hex} \cdot \frac{k_{H(op)\to H(cl)}}{k_{H(cl)\to H(op)} + k_{H(op)\to H(cl)}}, \; x_{oct}^{HF} = x_{oct} \cdot \frac{k_{O(cl)\to O(op)}}{k_{O(cl)\to O(op)} + k_{O(op)\to O(cl)}}$$

(Supplementary Equation 15)

and $x_{hex}^{HF} = x_{hex} \cdot \frac{k_{H(cl)\to H(op)}}{k_{H(cl)\to H(op)} + k_{H(op)\to H(cl)}}$.

For the mean rate constant $k_{MF*\to HF}$ it results:

$$\begin{aligned} k_{MF*\to HF} &= \left(1 + \frac{x_{hex}}{x_{oct}} \cdot \frac{k_{H(op)\to H(cl)} \cdot \left(k_{O(cl)\to O(op)} + k_{O(op)\to O(cl)}\right)}{k_{O(op)\to O(cl)} \cdot \left(k_{H(cl)\to H(op)} + k_{H(op)\to H(cl)}\right)}\right)^{-1} \\ &\quad \cdot \left(k_{O(cl)\to O(op)} - k_{H(cl)\to H(op)}\right) + k_{H(cl)\to H(op)} \\ &= \left(1 + \frac{K_{dis}^{O/H}}{[\mathrm{dim}]} \cdot \frac{k_{H(op)\to H(cl)} \cdot \left(k_{O(cl)\to O(op)} + k_{O(op)\to O(cl)}\right)}{k_{O(op)\to O(cl)} \cdot \left(k_{H(cl)\to H(op)} + k_{H(op)\to H(cl)}\right)}\right)^{-1} \\ &\quad \cdot \left(k_{O(cl)\to O(op)} - k_{H(cl)\to H(op)}\right) + k_{H(cl)\to H(op)} \end{aligned}$$

(Supplementary Equation 16)

and equivalently:

$$\begin{aligned} k_{HF\to MF*} &= \left(1 + \frac{K_{dis}^{O/H}}{[\mathrm{dim}]} \cdot \frac{k_{H(cl)\to H(op)} \cdot \left(k_{O(cl)\to O(op)} + k_{O(op)\to O(cl)}\right)}{k_{O(cl)\to O(op)} \cdot \left(k_{H(cl)\to H(op)} + k_{H(op)\to H(cl)}\right)}\right)^{-1} \\ &\quad \cdot \left(k_{O(op)\to O(cl)} - k_{H(op)\to H(cl)}\right) + k_{H(op)\to H(cl)} \end{aligned}$$

(Supplementary Equation 17)

Fitting to the data requires an estimation of the concentration of the free H2A-H2B-dimer, [dim]. Assuming only intact nucleosomes at the start of the experiment, each hexasome corresponds to one free H2A-H2B and each LF or D-only corresponds to two free H2A-H2B, [dim] can be approximated from the total concentration and the species fractions of the decomposition products as obtained by PDA and the fit of the kinetic model ([dynF], $\left[\mathrm{T}^{\mathrm{LF/NF}}\right]$ and $\left[\mathrm{DNA}^{\mathrm{NF}}\right]$ from PDA (**Table N2.1**), $\left[\mathrm{H}_{\mathrm{cl}}^{\mathrm{MF*}}\right]$ + $\left[\mathrm{H}_{\mathrm{op}}^{\mathrm{HF}}\right]$ iterative from fit in Fig. 4f, < 10% of total):

$$[\mathrm{dim}] = \left[\mathrm{H}_{\mathrm{cl}}^{\mathrm{MF*}}\right] + \left[\mathrm{H}_{\mathrm{op}}^{\mathrm{HF}}\right] + 2 \cdot \left(\left[\mathrm{T}^{\mathrm{LF/NF}}\right] + \left[\mathrm{DNA}^{\mathrm{NF}}\right]\right)$$

(Supplementary Equation 18)

Table N2.1. Total concentration and the species fractions of the decomposition products and empirical relation between [dim] and [nuc] (Fig. N2.1).

[nuc] **[pM]**	$\left[\mathrm{T}^{\mathrm{LF/NF}}\right]$ **[pM]**	$\left[\mathrm{DNA}^{\mathrm{NF}}\right]$ **[pM]**	[dynF] **[pM]**	$\left[\mathrm{H}_{\mathrm{cl}}^{\mathrm{MF*}}\right]$ + $\left[\mathrm{H}_{\mathrm{op}}^{\mathrm{HF}}\right]$ **[pM]**	$[\mathrm{dim}]_{\mathrm{PDA}}$ **[pM]**	$[\mathrm{dim}]_{\mathrm{fit}}$ from Fig. N2.1 **[pM]**
3	1.7	0.36	0.24	0.23	4.3	4.1
20	7	1.4	2.5	1.7	18	21
100	37	11	13	4.0	99	80
300	59	33	20	2.6	186	203
2000	135	217	75	1.6	706	1008
5000	488	975	152	1.3	2926	2187

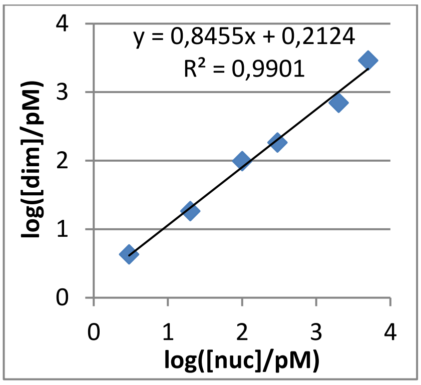

Fig. N2.1 Calibration of [dim]. The equilibrium concentration $[\text{dim}]_{\text{fit}}$ was determined by the iterative fit with Supplementary Equation 19. The obtained $[\text{dim}]_{\text{fit}}$ is listed in Table N2.1.

To reduce noise from the individual PDA-fits an empirical relation as

$$\log\left(\frac{[\text{dim}]}{\text{pM}}\right) = 0.8455\cdot\log\left(\frac{[\text{nuc}]}{\text{pM}}\right) + 0.2124, \quad R^2 = 0.99, \qquad \text{(Supplementary Equation 20)}$$

was obtained from a fit to the experimental data and used to convert the concentrations. Rate constants are taken as limiting values at lowest and highest dimer concentrations from the fit (rate constants vs. [dim], Fig. 4f). The obtained rate constants are compiled in Table 1. Equilibrium constants and relaxation times are calculated as:

$$K^{O}_{cl/op} = k_{O(cl)\to O(op)} / k_{O(op)\to O(cl)},\ t^{O}_{R} = (k_{O(cl)\to O(op)} + k_{O(op)\to O(cl)})^{-1},$$
$$K^{H}_{cl/op} = k_{H(cl)\to H(op)} / k_{H(op)\to H(cl)},\ t^{H}_{R} = (k_{H(cl)\to H(op)} + k_{H(op)\to H(cl)})^{-1}. \qquad \text{(Supplementary Equation 21)}$$

For the mean dissociation constant using Supplementary Equation 1 and $[\text{dim}] = 20\ \text{pM}$, one gets:

$$K^{O/H}_{dis} = \frac{[\text{H}]\cdot[\text{dim}]}{[\text{O}_{\text{dyn}}]} \approx 40\ \text{pM} = \frac{k_{O\to H}}{k_{H\to O}} \qquad \text{(Supplementary Equation 22)}$$

and for $k_{O\to H}$, assuming diffusion controlled association with $k^{diff}_{H\to O} \approx 10^{9}\,\text{M}^{-1}s^{-1}$ [5,6].

$$k_{O\to H} = K^{O/H}_{dis}\cdot k^{diff}_{H\to O} \approx 4\times10^{-11}\,\text{M}\times10^{9}\,\text{M}^{-1}\text{s}^{-1} = 0.04\,\text{s}^{-1}$$

$$\text{and}\quad t^{O/H}_{R} = \frac{1}{k_{O\to H} + k^{diff}_{H\to O}\cdot[\text{dim}]} = \frac{1}{k^{diff}_{H\to O}}\cdot\frac{1}{K^{O/H}_{dis} + [\text{dim}]} \approx 17\,\text{s} \qquad \text{(Supplementary Equation 23)}$$

Since we observed $t^{O/H}_{R} < 300\,\text{s}$ (average time needed for sample preparation before measurements start) the estimated association rate is not more than 10x smaller than would be expected for a diffusion controlled process.

Step I: The static octasome - dynamic octasome transition

Assuming an equilibrium $\text{O}^{\text{MF}}_{\text{st}} \rightleftarrows \text{O}^{\text{MF*}}_{\text{cl}}$ (Fig. 8, red box) decoupled from $\text{O}_{\text{dyn}} \rightleftarrows \text{H} + \text{dim}$ and $\text{MF*} \rightleftarrows \text{HF}$ (black box) due to kinetics on different time scales, the equilibrium constant

$K^{O}_{st/cl} = \left[\mathrm{O}^{\mathrm{MF*}}_{\mathrm{cl}}\right]/\left[\mathrm{O}^{\mathrm{MF}}_{\mathrm{st}}\right]$ is given by the corresponding species fractions determined by PDA analysis. A value of $K^{O}_{st/cl} = 0.06 \pm 0.01$ was found, independent of nucleosome concentration. With $K^{O}_{st/cl} = k_{O(st)\to O(cl)} / k_{O(cl)\to O(st)}$ being the ratio and the lower limit for the relaxation time (as estimated by simulation) $t_R^{O(st/cl)} = (k_{O(st)\to O(cl)} + k_{O(cl)\to O(st)})^{-1} > 3\ \mathrm{ms}$ the inverse sum of the involved rate constants the upper limits $k_{O(st)\to O(cl)} < 0.02\ \mathrm{ms}^{-1}$ and $k_{O(cl)\to O(st)} < 0.32\ \mathrm{ms}^{-1}$ are obtained.

The relaxation time for the $\mathrm{O}^{\mathrm{MF*}}_{\mathrm{cl}} \rightleftarrows \mathrm{O}^{\mathrm{MF}}_{\mathrm{st}}$ interconversion has to be of the order of the diffusion time or longer to allow the static and dynamic octasome species to appear separated in a single molecule experiment.

Step V: Disassembly of hexasome

This process is very slow but eviction of the second H2A-H2B dimer can be promoted by increased salt concentration. The global fit of decays measured by time evolution of mid/high FRET species (octasome and hexasome) and low FRET species (open structure and free DNA) (Supplementary Fig. 4a-d) yields a dissociation rate constant $k_{H(op)\to T}$. The data were analyzed as follows.

Overall signal change due to adsorption/desorption/bleaching etc. is accounted for by approximating a linear behavior of the total number of bursts per histogram bin:

$$N_{tot}(t) = N_{tot,0} \cdot (1 + k_{tot} \cdot t) \qquad \text{(Supplementary Equation 24)}$$

The data sets were divided into two subgroups: LF ($7.5 < F_D/F_A$) represents fully dissociated nucleosomes, while (MF+*dyn*F+HF) populations ($F_D/F_A < 7.5$) were combined to represent (sub-) nucleosomal particles at various stages of disassembly. The change of each population with time was approximated by the following model:

$$N_{MF}(t) = N_{tot,0} \cdot \left(A \cdot e^{-k_{H(op)\to T} \cdot t} + \gamma \cdot \left(1 - A \cdot e^{-k_{H(op)\to T} \cdot t}\right)\right) \cdot (1 + k_{tot} \cdot t) \qquad \text{(Supplementary Equation 25)}$$

$$N_{LF}(t) = N_{tot,0} \cdot (1 - \gamma) \cdot \left(1 - A \cdot e^{-k_{H(op)\to T} \cdot t}\right) \cdot (1 + k_{tot} \cdot t) \qquad \text{(Supplementary Equation 26)}$$

and

$$N_{tot}(t) = N_{MF}(t) + N_{LF}(t) = N_{tot,0} \cdot (1 + k_{tot} \cdot t) \qquad \text{(Supplementary Equation 27)}$$

Incomplete separation of the species due to overlap of the individual distributions leading to LF-bursts in the MF-fraction is accounted for by a crosstalk term γ. γ was estimated by fitting lognormal functions to 1dimensional (1D) F_D/F_A histograms of the samples and led to a value of $\gamma \approx 0.05$. k_{tot} was determined first by fitting Supplementary Equation 23 to 1D histograms of N_{tot} vs. t (not shown). We have found $k_{tot} \approx \pm(10^{-5}\ \text{to}\ 10^{-6})\mathrm{s}^{-1}$. In the second step Supplementary Equations 24-25 were fitted globally to the time decays of two fractions (Supplementary Fig. 4a-d).

Results as function of NaCl concentration are presented in Supplementary Fig. 4e. As one can notice, the $k_{H(op)\to T}$ values differ from zero even at very low salt concentration. In order to estimate this limiting value, the data have been fitted to the following equation:

$$k_{H(op)\to T} = k^{0}_{H(op)\to T} + k^{0'}_{H(op)\to T} \cdot e^{[\mathrm{NaCl}]/c}\ \mathrm{s}^{-1} \qquad \text{(Supplementary Equation 28)}$$

where $k^{0}_{H(op)\to T}$ represents the off-rate constant at zero salt. The fit yields the following values:

$k^{0}_{H(op)\to T} = (1.8 \pm 0.5)\times 10^{-5}\ \mathrm{s}^{-1}$, $k^{0'}_{H(op)\to T} = (3.4 \pm 4.9)\times 10^{-8}\ \mathrm{s}^{-1}$ and $c = (117 \pm 17)\,\mathrm{mM}$.

Let us now estimate the association rate constant. Ignoring intact nucleosomes (octasomes), the last reaction can be approximated as $H \rightleftarrows T + \dim$. Under equilibrium conditions we can write:

$$K_{dis}^{H/T} = \frac{[\mathrm{T}]\cdot[\mathrm{dim}]}{[\mathrm{H}]} = \frac{[\mathrm{T}]^2}{[\mathrm{H}]} = \frac{[\mathrm{T}]^2}{[\mathrm{total}]-[\mathrm{T}]}, \quad \text{(Supplementary Equation 29)}$$

with

$$[\mathrm{total}] = [\mathrm{H}] + [\mathrm{T}] = [\mathrm{H}] + [\mathrm{dim}]$$

which easily can be transformed into $[\mathrm{T}]^2 + K_{dis}^{H/T}\cdot[\mathrm{T}] - K_{dis}^{H/T}\cdot[\mathrm{total}] = 0$ and solved in terms of the dissociation degree, $\alpha = \frac{[\mathrm{T}]}{[\mathrm{total}]}$:

$$\alpha = \frac{K_{dis}^{H/T}}{2\cdot[\mathrm{total}]}\left(\sqrt{1+\frac{4\cdot[\mathrm{total}]}{K_{dis}^{H/T}}}-1\right). \quad \text{(Supplementary Equation 30)}$$

The dissociation degree α as a function of $[\mathrm{total}]/K_{dis}^{H/T}$ ratio has been simulated and presented as graph in Fig. N2.2. Assuming a diffusion controlled association rate as for step III we would get:

$K_{dis}^{H/T} = \frac{k_{H(op)\to T}}{k^{diff}_{T\to H(op)}} \approx \frac{1.8\times 10^{-5}\ \mathrm{s}^{-1}}{10^{9}\ \mathrm{M}^{-1}\mathrm{s}^{-1}} = 0.018\,\mathrm{pM}$ and $t_R^{H/T} = \frac{1}{k_{H(op)\to T} + k^{diff}_{T\to H(op)}\cdot[\mathrm{dim}]} \approx 50\,\mathrm{s}$ at 20 pM dimer concentration. Since the observed relaxation time at [dim] ≈ [nuc] = 20 pM ($\alpha > 0.3$) is orders of magnitude larger, $t_R^{H/T} > 5000\,\mathrm{s}$, we can conclude that the association is much slower than a purely diffusion controlled process: $k_{T\to H(op)} < 0.01\cdot k^{diff}_{T\to H(op)}$. This can be rationalized in view of the much larger structural changes involved in step V as compared to step III.

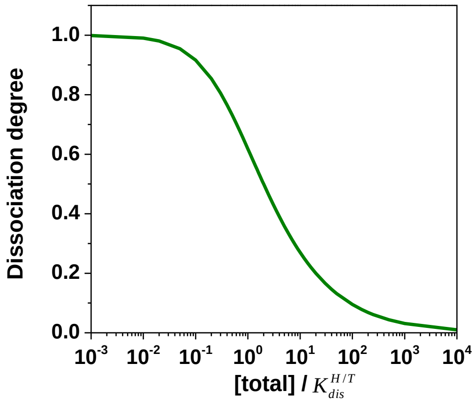

Fig. N2.2. Dissociation degree α. Simulation of dissociation degree α as a function of total nucleosome concentration [total] and dissociation equilibrium constant $\mathrm{K_{dis}^{H/T}}$ ratio, $[\mathrm{total}]/\mathrm{K_{dis}^{H/T}}$.

Comparison with recently published results

Kim et al. do not resolve step I and II in their study (no separation of MF and MF*)[7]. The corresponding constants (measured at 100 mM NaCl) as extracted from their paper are listed in **Table N2.2**. A comparison with our results requires the estimation of rate constants for the average process.

Table N2.2: Corresponding rate constants (measured at 100 mM NaCl) as extracted from paper Kim et al. : they do not resolve step I and II in their study (no separation of MF and MF*)[7].

**Step**	**Rate constants**		**Equilibrium constants**		**Relaxation times**	
I & II	$k_{O(cl)\to O(op)}$<br>$k_{O(op)\to O(cl)}$	(0.28±0.03) $10^3 s^{-1}$<br>(0.49±0.07)·$10^3 s^{-1}$	$K_{cl/op}^{O(I\&II)}$	0.57±0.1	$t_R^{O(cl/op)}$	(1.3±0.2) ms

Thus combining step I&II (our data, 150 mM NaCl) yields:

$$K_{cl/op}^{O} = \frac{\left[O_{op}^{HF}\right]}{\left[O_{cl}^{MF*}\right]}; \quad K_{st/cl}^{O} = \frac{\left[O_{cl}^{MF*}\right]}{\left[O_{st}^{MF}\right]}$$

$$K_{cl/op}^{O(I\&II)} = \frac{\left[O_{op}^{HF}\right]}{\left[O_{cl}^{MF*}\right]+\left[O_{st}^{MF}\right]} = \frac{\left[O_{op}^{HF}\right]}{\left[O_{cl}^{MF*}\right]\left(1+\frac{1}{K_{st/cl}^{O}}\right)} = K_{cl/op}^{O}\left(1+\frac{1}{K_{st/cl}^{O}}\right)^{-1} \qquad \text{(Supplementary Equation 31)}$$

$$= 1.56\cdot\left(1+\frac{1}{0.06}\right)^{-1} = 0.09 \pm 0.02$$

Kim et al. report slower kinetics by more than an order of magnitude and a different position of the equilibrium, possibly due to surface effects and different salt conditions.

In analogy with Kim et al. the potential energy scheme of the octasome as estimated from our obtained equilibrium constants involving three states was estimated and is shown in Fig. N2.3.

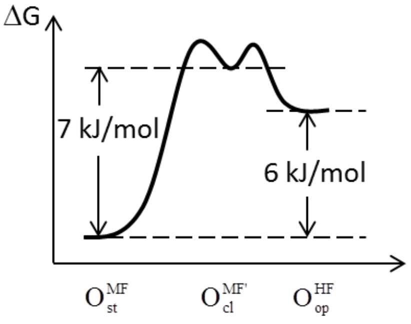

Fig. N2.3. Potential energy landscape of octasome species. Sketch for the estimated potential energy landscape for the three observed octasome states.

Supplementary Note 3: Parameterization of the geometric model for $I_\beta I_\alpha$-nucleosomes

We tried to correlate the kinetic scheme with structural candidates for (sub)nucleosomal particles, using a simplified geometric model of the nucleosome (Fig. N3.1). The model was parameterized based on the crystal structure (PDB ID: 3LZ1) and simulation of accessible fluorophore space in $I_\beta I_\alpha$ nucleosomes to determine distances between the mean positions of donor and acceptor, R_{mp}, [4]. Note, that $R_{DA,mp}$ cannot measured experimentally [4]. Therefore R_{mp} is converted to FRET-average mean distance $\langle R_{DA}\rangle_E$ by the following polynomial: $\langle R_{DA}\rangle_E = 20.498 + 0.4778\ R_{mp} + 0.0030095\ (R_{mp})^2$ - $0{,}0000016467\ (R_{mp})^3$. The computed distance for the intact nucleosome is R_{mp} = 62 Å, the corresponding $\langle R_{DA}\rangle_E$ = 61.3 Å being in excellent agreement with our average experimental data $\langle R_{DA}{}^{MF}\rangle_E$ = (61.1 ± 3.0) Å.

Choice of the template structure. Earlier on we had proposed a step-wise disassembly through loss of H2A-H2B dimers[8], but the lack of dynamic PDA at that time precluded its detailed kinetic analysis. The dynamic intermediate (here dynF) was treated as a static species (HF in [8]), whose apparent inter-dye distance agreed with R_{DA} estimated from the widely used nucleosome crystal structure (PDB ID: 1KX5), and was erroneously assigned to the intact nucleosome. The 1KX5 structure, however, features a DNA sequence different from the 601 used in our nucleosomes, and thus did not offer exact positional information. Assuming that the center of our DNA coincided with the dyad axis in 1KX5, it has led to an underestimated R_{DA} for the intact nucleosome. A high-resolution crystal structure for 601 nucleosomes was only recently published (PDB ID: 3LZ1) [9] and showed a 6 bp shift of our DNA fragment center relative to the dyad axis. The FRET distance modeled with this structure now agrees much better with measured R_{MF}, and the improved PDA analysis clearly identified the dynamic character of dynF. Thus, our current assignment of the intact nucleosome to MF is correct and should not be confused with our earlier notation.

Geometric modelling. As shown in Fig. N3.1, the nucleosome is approximated as a cylindrical disc of effective diameter R_{core} = 3.3 nm. The inner turn of nucleosomal DNA is slightly more compact than the outer turn with about 78 bp per full turn, giving an average superhelical shift of $d\varphi$ = 4.6° per bp DNA. The diameter of DNA was taken to be d_{DNA} = 2 nm. Centers of mass of the fluorophore's accessible space were used to represent effective dye positions. Effective radii and angular positions (as counted in base pairs from the dyad axis) were R_A = 5.3 nm and x_A = -46.5 for the acceptor and R_D = 5.4 nm and x_D = 47.5) for the donor. The effective positions of the two dimer:tetramer interfaces were taken as x_h = ±31. The axial displacement of the dyes is d_z = 1.8 nm per superhelical turn.

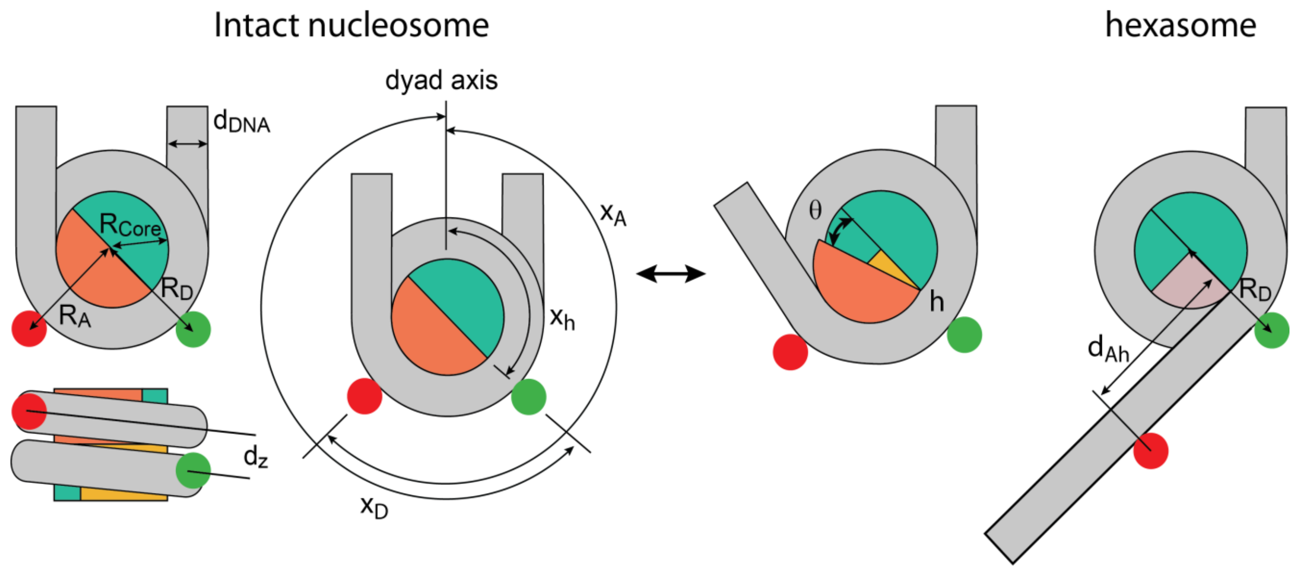

Fig. N3.1. Estimation of interdye distances by geometric computation. Parameterization of the geometric model for $I_{\beta}I_{\alpha}$-nucleosomes: Values were estimated from the crystal structure 3LZ1 and calculated accessible space for each fluorophore (r.ed and green clouds in Fig. 2a in the main text): d_{DNA} = 20 Å, R_{core} = 33 Å. R_A = 53 Å, x_A = -46.5, R_D = 54 Å, x_D = 47.5, x_h = ±31, d_z = 18 Å. For the hexasome structures: d_{Dh} = 53.6 Å (not shown) and d_{Ah} = 50.3 Å. For estimation of possible HF structures, the opening of the dimer:tetramer interface around a hinge point h is parameterized by the opening angle θ. The (H3-H4)$_2$ tetramer is shown in blue-green, while both H2A-H2B heterodimers are shown in orange and dark yellow.

To model the hexasome we removed one of the two H2A-H2B heterodimers and allowed the DNA arm to exit tangentially from the broken dimer:tetramer interface. The distance between the fluorophore on the open DNA and the broken interface was computed as $d_{Dh/Ah}$ = $((x_{D/A} - x_h)/10.5)*3.41$ nm with a helical pitch of undisturbed B-DNA of 3.41 nm and 10.5 base pairs per helical turn. In all simulations it was assumed that the superhelical pitch remains the same for wrapped and unwrapped DNA.
To compute the donor-acceptor distance during opening of the dimer:tetramer interface, we assumed that dimer and tetramer open up at a hinge point located at the interface between DNA and the protein core. The distance of the hinge point from the center was taken to be the same as the radius of the histone core, R_{core}. For simplicity, distortion of the DNA path at larger opening angles was ignored.
Inter-dye distances were calculated from standard geometric considerations. Further detailed drawings (Fig. N3.2) and the source code of the simulations for the programming tool IGOR Pro (WaveMetrics, Lake Oswego, OR, USA) is provided below.

Definitions and list of variables

- Nucleosome is approximated as a cylindrical disc of effective diameter $\mathbf{R_{core}}$ = 3.3 nm.
- The inner turn of nucleosomal DNA is slightly more compact than the outer turn with about 78 bp per full turn, giving an average superhelical shift of $\mathbf{d\varphi}$ = 4.6° per bp DNA.
- The diameter of DNA was taken to be $\mathbf{d_{DNA}}$ = 2 nm.
- Effective radii and angular positions (as counted in base pairs from the dyad axis) were
- $\mathbf{R_A}$ = 5.3 nm and $\mathbf{x_A}$ = -46.5 for the acceptor and
- $\mathbf{R_D}$ = 5.4 nm and $\mathbf{x_D}$ = 47.5 for the donor.
- The effective positions of the two dimer:tetramer interfaces were taken as $\mathbf{x_h}$ = ±31.

- The axial displacement of the dyes is $\mathbf{d_z}$ = 1.8 nm per superhelical turn.
- To **model the hexasome** we removed one of the two H2A-H2B heterodimers and allowed the DNA arm to exit tangentially from the broken dimer:tetramer interface.
- The distance between the fluorophore on the open DNA and the broken interface was computed as
- $\mathbf{d_{Dh/Ah}} = ((\mathbf{x_{D/A}} - \mathbf{x_h})/10.5)*3.41$ nm
- with a helical pitch of undisturbed B-DNA of **3.41 nm** and **10.5** base pairs per helical turn.
- In all simulations it was assumed that the superhelical pitch remains the same for wrapped and unwrapped DNA.

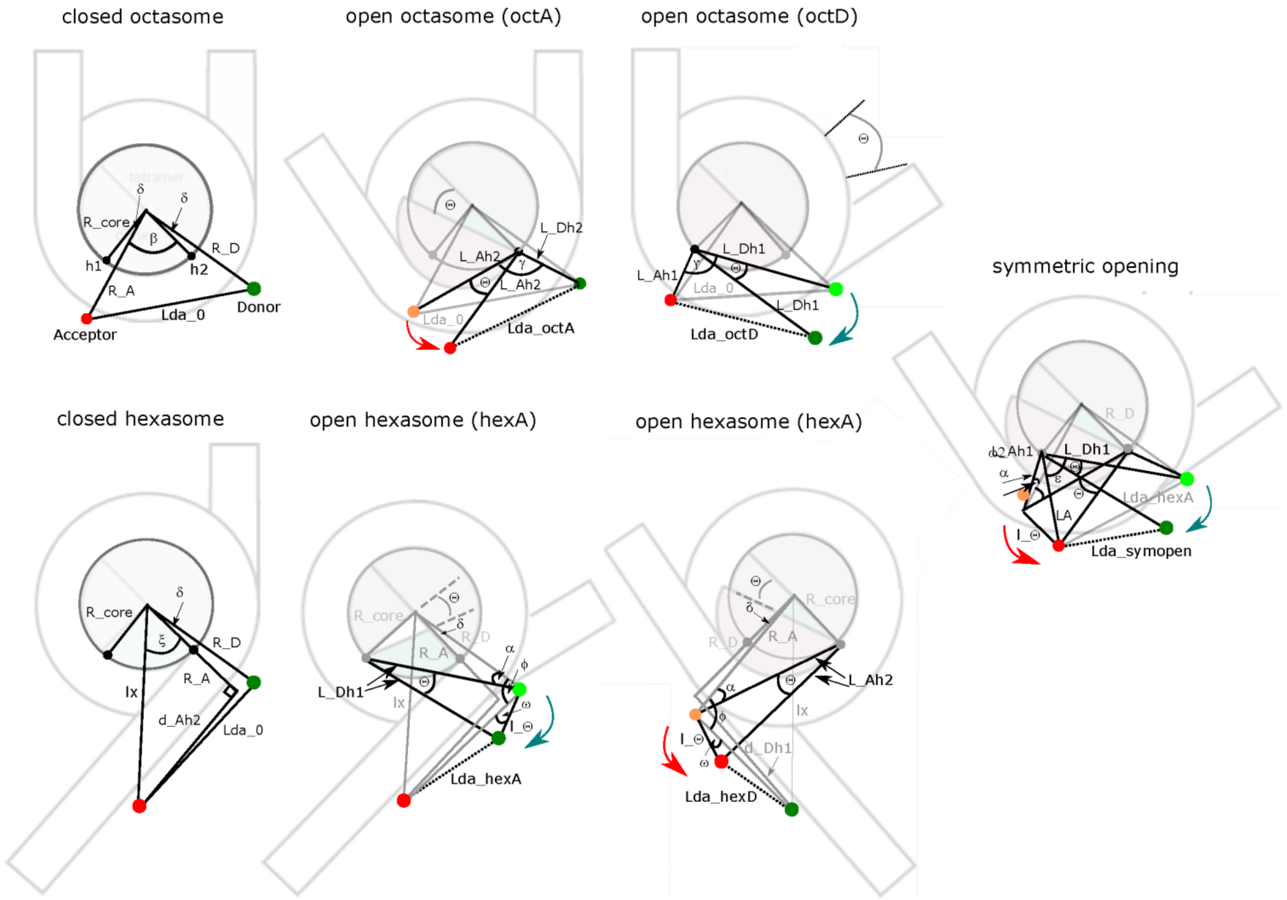

Fig. N3.2. Definitions for the geometric computation.

Simulation code for the programming tool IGOR Pro (WaveMetrics, Lake Oswego, OR, USA)

```
macro geomodel()

  // geometric parameters

   Variable R_D=5.4
   Variable R_A=5.3
   Variable x_A=46.5
   Variable x_D=47.5
   Variable x_h=31
```

```
Variable dz=1.8
Variable R_core=3.3
Variable bpperturn=78

// angles and distances

Variable alpha=0
Variable beta=0
Variable gamma=0
Variable xi=0
Variable delta=0
Variable omega2=0
Variable Lz = 0
Variable Lda_0=0
Variable Rda_0=0
Variable lx=0
Variable L_Dh1=0
Variable L_Ah1=0
Variable L_Dh2=0
Variable L_Ah2=0
Variable d_Ah2=0
Variable d_Dh2=0

// calculate z-component of interdye distance

 Lz=(x_D+x_A)/bpperturn*dz

// opening angle between dimer and tetramer (in degrees)

 Make /O/D/N=100 theta
 theta=p

// define angle-dependent xy-components of interdye distances

 Make /O/D/N=100 Lda_octA
 Make /O/D/N=100 Lda_octD
 Make /O/D/N=100 Lda_hexA
 Make /O/D/N=100 Lda_hexD
 Make /O/D/N=100 Lda_symopen

// define angle-dependent 3D interdye distances

 Make /O/D/N=100 Rda_octA
 Make /O/D/N=100 Rda_octD
 Make /O/D/N=100 Rda_hexA
 Make /O/D/N=100 Rda_hexD
 Make /O/D/N=100 Rda_symopen

// additional angle-dependent parameters

 Make /O/D/N=100 l_theta
 Make /O/D/N=100 LA
 Make /O/D/N=100 omega
 Make /O/D/N=100 epsilon

// intact nucleosome (theta = 0)

 beta= (1-(bpperturn-x_A+x_h)/bpperturn)*360
 delta=(x_D+x_h-bpperturn)/bpperturn*360
 Lda_0=sqrt(R_D^2+R_A^2-2*R_D*R_A*cos((beta+delta)/180*pi))
 Rda_0=sqrt(Lda_0^2+Lz^2)

// octasome, breathing A-dimer
```

```
 L_Dh2=sqrt(R_D^2+R_core^2-2*R_D*R_core*cos(delta/180*pi))
 L_Ah2=sqrt(R_A^2+R_core^2-2*R_A*R_core*cos(beta/180*pi))
 gamma=acos((L_Dh2^2+L_Ah2^2-Lda_0^2)/(2*L_Dh2*L_Ah2))*180/pi
 Lda_octA=sqrt(L_Dh2^2+L_Ah2^2-2*L_Dh2*L_Ah2*cos((gamma-theta)/180*pi))
 Rda_octA=sqrt(Lda_octA^2+Lz^2)

// octasome, breathing D-dimer

 L_Dh1=sqrt(R_D^2+R_core^2-2*R_D*R_core*cos((beta+2*delta)/180*pi))
 L_Ah1=sqrt(R_A^2+R_core^2-2*R_A*R_core*cos(delta/180*pi))
 gamma=acos((L_Dh1^2+L_Ah1^2-Lda_0^2)/(2*L_Dh1*L_Ah1))*180/pi
 Lda_octD=sqrt(L_Dh1^2+LAh1^2-2*L_Dh1*LAh1*cos((gamma-theta)/180*pi))
 Rda_octD=sqrt(Lda_octD^2+Lz^2)

// hexasome, A-dimer lost, breathing D-arm

 d_Ah2=(x_A-x_h)/10.5*3.41
 lx=sqrt(R_A^2+d_Ah2^2)
 xi=90-atan(R_A/d_Ah2)*180/pi
 Lda_0=sqrt(R_D^2+lx^2-2*R_D*lx*cos((xi+delta)/180*pi))
 alpha=acos((R_core^2-R_D^2-L_Dh1^2)/(2*R_D*L_Dh1))*180/pi
 phi=acos((lx^2-Lda_0^2-L_R_D^2)/(2*Lda_0*R_D))*180/pi
 l_theta=2*L_Dh1*sin(0.5*theta/180*pi)
 omega=0.5*(180-theta)-(phi-alpha)
 Lda_hexA=sqrt(Lda_0^2+l_theta^2-2*Lda_0*l_theta*cos(omega/180*pi))
 Rda_ hexA=sqrt(Lda_ hexA^2+Lz^2)

// hexasome, D-dimer lost, breathing A-dimer

 d_Dh1=(x_D-x_h)/10.5*3.41
 lx=sqrt(R_D^2+d_Dh1^2)
 xi=90-atan(R_D/d_Dh1)*180/pi
 Lda_0=sqrt(R_A^2+lx^2-2*R_A*lx*cos((xi-delta)/180*pi))
 l_theta=2*L_Ah2*sin(0.5*theta/180*pi)
 theta=acos((R_core^2-R_A^2-L_Ah2^2)/(2*R_A*L_Ah2))*180/pi
 phi=acos((lx^2-Lda_0^2-L_R_A^2)/(2*Lda_0*R_A))*180/pi
 omega=0.5*(180-theta)-(phi-alpha)
 Lda_hexD=sqrt(Lda_0^2+l_theta^2-2*Lda_0*l_theta*cos(omega/180*pi))
 Rda_ hexD=sqrt(Lda_ hexD^2+Lz^2)

// simultaneous opening

omega2=acos((R_core^2-R_A^2-L_Ah1^2)/(2*R_A*L_Ah1))*180/pi
LA=sqrt(l_theta^2+L_Ah1^2-2*L_Ah1*l_theta*cos((omega2+alpha+90-0.5*theta)*pi/180))
epsilon=acos((Lda_octA^2-LA^2-L_Dh1^2)/(2*LA*L_Dh1))*180/pi
Lda_symopen=sqrt(LA^2+L_Dh1^2-2*LA*L_Dh1*cos((epsilon-theta)*pi/180))
Rda_symopen=sqrt(Lda_symopen^2+Lz^2)

End
```

Supplementary Note 4: Species-selective filtered FCS

To determine the additional time scales of conformational fluctuations in $I_\beta I_\alpha$-wt nucleosomes we performed filtered FCS on the smFRET data as described in ref. 22 and 25 in the main text. Species-selective lifetime filters for the HF and LF species were first computed via stacked TAC histograms of green and red detection channels:

We first computed appropriate lifetime patterns for the LF and HF subspecies by applying time window analysis to the smFRET data. Due to fast nucleosome dynamics, the HF signature is mostly hidden within the dynF population. Thus, instead of analyzing data on the level of whole bursts, we regrouped photons from each burst into smaller time windows (TW) of equal duration (TW = 1 ms). A 2D histogram of all 1 ms time windows is then used to identify LF and HF subspecies for lifetime filter generation. Supplementary Fig. 5 a, b compare 2D histograms of FRET efficiency E versus $\langle\tau_D\rangle_F$ (average donor lifetime in presence of acceptor) for burstwise and time window analysis. As one can notice, the HF (red box) and LF regions (blue box) are more populated in the case of individual time windows, compared to the burstwise distribution. Fluorescence lifetime decays for HF and LF species (Supplementary Fig. 5 c, d) are generated from TW's in the red and blue boxes as described in ref. 25 in the main text. The scatter contribution is always taken into account via decay patterns of buffer (gray histograms in Supplementary Fig. 5 c, d). The generated filters are plotted for stacked parallel and perpendicular green-red channels in Supplementary Fig. 5 e, f. All analyses were carried out by a LabView based, custom designed software package: http://www.mpc.hhu.de/software/software-package.html.

Species auto- and cross-correlation functions (SACF and SCCF) were generated and fitted globally: Model functions combining four bunching (in SACF) and four anti-correlating terms (in SCCF) with a 3D Gaussian diffusion term were used to fit experimental curves.

SACF:

$$G(t_c) = offset + \left(1+\frac{t_c}{t_d}\right)^{-1}\cdot\left(1+\left(\frac{\omega_0}{z_0}\right)^2\cdot\frac{t_c}{t_d}\right)^{-\frac{1}{2}}\cdot\left(1-|T|+|T|\cdot e^{-\left(\frac{t_c}{|t_T|}\right)}\right)$$
$$\cdot\left(1-|X_{R1}|+|X_{R1}|\cdot e^{-\left(\frac{t_c}{|t_{R1}|}\right)}-|X_{R2}|+|X_{R2}|\cdot e^{-\left(\frac{t_c}{|t_{R2}|}\right)}-|X_{R3}|+|X_{R3}|\cdot e^{-\left(\frac{t_c}{|t_{R3}|}\right)}-|X_{R4}|+|X_{R4}|\cdot e^{-\left(\frac{t_c}{|t_{R4}|}\right)}\right)$$

SCCF: (Supplementary Equation 32)

$$G(t_c) = offset + \left(1+\frac{t_c}{t_d}\right)^{-1}\cdot\left(1+\left(\frac{\omega_0}{z_0}\right)^2\cdot\frac{t_c}{t_d}\right)^{-\frac{1}{2}}\cdot\left(1-|T|+|T|\cdot e^{-\left(\frac{t_c}{|t_T|}\right)}\right)\cdot\left(1-|B|\cdot e^{-\left(\frac{t_c}{|t_B|}\right)}\right)$$
$$\cdot\left(1-|AC|\cdot\left(|x_{R1}|\cdot e^{-\left(\frac{t_c}{|t_{R1}|}\right)}+|x_{R2}|\cdot e^{-\left(\frac{t_c}{|t_{R2}|}\right)}+|x_{R3}|\cdot e^{-\left(\frac{t_c}{|t_{R3}|}\right)}+\left|1-|x_{R1}|-|x_{R2}|-|x_{R3}|\right|\cdot e^{-\left(\frac{t_c}{|t_{R4}|}\right)}\right)\right)$$

where spatial distribution of the detection probabilities are $w(x,y,z)=\exp\left(-2\left(x^2+y^2\right)/\omega_0^2\right)\exp\left(-2z^2/z_0^2\right)$. The $1/e^2$ radii in x, y and in z directions are denoted by ω_0 and z_0, respectively. X_{Ri} and x_{Ri} are representing amplitudes of bunching and fractions of anticorrelation terms, correspondingly. AC is the total amplitude of anticorrelated terms. t_{Ri} are representing relaxation times. The triplet amplitude and time are denoted as T and t_T, respectively [2].

All four relaxation times, the diffusion time and the focus shape parameter (z_0/ω_0) were global parameters for all filtered FCS curves.

Experimental SACF and SCCF curves with corresponding fits are shown in Supplementary Fig. 5 g, h for 20 pM $I_\beta I_\alpha$-wt nucleosomes at 500 mM NaCl. The 3 relevant time scales are marked by gray bars.

Supplementary Note 5: Characterization of nucleosome disassembly by salt-induced destabilization using ensemble FRET measurements

Quantification of ensemble FRET measurements using a scanning microplate-reader.

A variable mode scanner (Typhoon 9400, GE Healthcare) was used to measure the proximity ratio of samples in 384-well microplates [10]. All images were acquired with a pixel resolution of 100 µm and the focus set 3 mm above the scanner surface, placing the imaging plane inside the microplate array. Fluorescence was recorded on two photomultiplier tubes (PMT) with voltages and filter settings as follows:

- donor channel (I_D^{Dex}): excitation at 488 nm, detection at 500-540 nm; PMT voltage 625 V.
- acceptor channel (I_A^{Aex}): excitation at 532 nm, detection at 595-625 nm; PMT voltage 675 V.
- transfer channel (I_A^{Dex}): excitation at 488 nm, detection at 595-625 nm, PMT voltage 675 V.

The proximity ratio P (eq. 1 Methods of the main text) was estimated from measured donor and acceptor raw intensities I_D^{Dex} and I_A^{Dex} upon donor excitation. These contain additional contributions from background (B_D^{Dex} and B_A^{Dex}), donor crosstalk into the acceptor channel (α_{DT}) and direct excitation of the acceptor dye at 488 nm (f_{dir}), which have to be corrected for.

$$\begin{aligned}\left(I_A^{Dex}\right)_{corr} &= \left(I_A^{Dex} - B_A^{Dex}\right) - \alpha_{DT}\left(I_D^{Dex} - B_D^{Dex}\right) - f_{dir} \\ \left(I_D^{Dex}\right)_{corr} &= I_D^{Dex} - B_D^{Dex}\end{aligned} \quad \text{(Supplementary Equation 33)}$$

Background. The average background signal per well, B_D^{Dex}, B_A^{Dex} and B_A^{Aex}, is measured in one or more separate wells containing pure buffer solution.

Crosstalk of donor emission into the acceptor channel. Three wells were filled with a donor-only sample and measured in parallel to double-labeled FRET samples. From the intensities in the donor and acceptor channel upon excitation with 488 nm we obtained the crosstalk factor as

$$\alpha_{DT} = \left(\frac{I_A^{Dex} - B_A^{Dex}}{I_D^{Dex} - B_D^{Dex}}\right)_{D-only} . \quad \text{(Supplementary Equation 34)}$$

Direct excitation of the acceptor dye. f_{dir} was determined from a separate acceptor-only sample that was loaded on the same plate as the FRET samples. Acceptor-only and FRET samples were probed with two excitation wavelengths; excitation with 532 nm selectively excited all acceptor molecules, yielding acceptor intensities $(I_A^{Aex})_{\text{A-only}}$ and $(I_A^{Aex})_{\text{FRET}}$, while excitation with 488 nm yielded intensities $(I_A^{Dex})_{\text{A-only}}$ and $(I_A^{Dex})_{\text{FRET}}$.

After background correction the only contribution to $(I_A^{Dex})_{\text{A-only}}$ arose from direct acceptor excitation and we define a ratio

$$S_A = \left(\frac{I_A^{Dex} - B_A^{Dex}}{I_A^{Aex} - B_A^{Aex}}\right)_{A-only} . \quad \text{(Supplementary Equation 35)}$$

S_A is assumed to be the same for acceptor molecules in the FRET samples and in the acceptor-only sample. Based on the measured intensities $(I_A^{Aex})_{\text{FRET}}$, we computed the contribution of direct excitation in each FRET sample as

$$f_{dir} = S_A \cdot \left(I_A^{Aex} - B_A^{Aex}\right)_{FRET} . \quad \text{(Supplementary Equation 36)}$$

Fit function for data analysis.

Data were plotted as a function of NaCl concentration in mM and approximated by a sigmoid function to determine the NaCl midpoint concentration $c_{1/2}$ where 50% of the change in P has occurred:

$$P([NaCl]) = P(0) + \frac{P(\infty) - P(0)}{1 + \exp((c_{1/2} - [NaCl])/b)} \quad \text{(Supplementary Equation 37)}$$

$$\frac{dP}{d[\mathrm{NaCl}]} = (P(\infty) - P(0)) \frac{\exp((c_{1/2} - [\mathrm{NaCl}])/b)}{b(1 + \exp((c_{1/2} - [\mathrm{NaCl}])/b))^2} = \frac{P(\infty) - P(0)}{4b} \quad \text{for} \quad [\mathrm{NaCl}] = c_{1/2} \quad \text{(Supplementary Equation 38)}$$

$P(0)$ and $P(\infty)$ are maximum amplitude and offset of the fit curve, $c_{1/2}$ is the midpoint of the curve and b is proportional to the inverse slope, dP/d[NaCl] (Supplementary Equation 37), at [NaCl] = $c_{1/2}$.as a measure for the half-width of the transition. The values of $c_{1/2}$ and b are compiled for all fits in Supplementary Table 4.

Comparison of ensemble and single-molecule studies.

In contrast to the PIE-MFD derived $c_{1/2}$ values, those obtained from ensemble FRET characterize not purely the proportion of intact particles but a FRET weighted average of all FRET-active species. Additionally, the $c_{1/2}$ values derived from species selective PIE-MFD and from ensemble measurements differ slightly because we probed different regions within the nucleosome. Nevertheless, their side specific differences indicate that both moving off the DNA ends and dimer eviction proceed preferentially from the α-side.

Supplementary Note 6: Analysis of the donor and acceptor mobility in MF/MF* via fluorescence anisotropy

Fluorophore mobility in the MF/MF* subpopulation was assessed by species-selective anisotropy. As presented in Supplementary Table 4, the acceptor clearly showed two anisotropies. At pM concentrations mostly low anisotropies were observed, indicating unhindered acceptor motion due to lack of the nearby histone dimer. At nanomolar concentrations, a second species with larger anisotropy (restricted dye mobility) became dominant: a conformation in which the acceptor still feels the presence of the neighboring heterodimer. Notably, donor anisotropies remained fairly insensitive to nucleosome concentration with a single component of r_1 = 0.11-0.14. This suggests that in MF/MF* the H2A-H2B heterodimer next to the donor (D_β) remains in place at all nucleosome concentrations analyzed in this work.

Supplementary Methods

DNA sequence and nucleosome labeling

The sequence of the 170 bp DNA with fluorophore positions as used here is shown below (donor in green and acceptor in red). Bases in brackets were replaced with thymine for labeling. The center of the fragment is marked with an asterisk (*).

Forward strand:

5`-T$^{E\alpha}$(C)ATGC ACAGGATGTA TATATCTGAC ACGTGCC(-53)**T**$^{I\alpha}$GG AGACTAGGGA GTAATCCCCT TGGCGGTTAA

AACGC(-15)T $^{Dy\alpha}$(G)GGGG ACAGCGCGTA * CGTGCGTTTA AGCGGTGCTA GAGCTGTCTA CGACCAATTG

AGCGGCCTCG GCACCGGGAT TCTCCAGGGC GGCCGCGTAT AGGGT-3'

Reverse strand:

3`-GTACG TGTCCTACAT ATATAGACTG TGCACGGA CC TCTGATCCCT CATTAGGGGA ACCGCCAATT

TTGCG CCCCC TGTCGCGCAT * GCACGCAAAT TCGC(C)(+15)T $^{Dy\beta}$ACGAT CTCGACAGAT GCTGGTTAAC

(+41)T$^{I\beta}$ CGCCGGAGC CGTGGCCCTA AGAGGTCCCG CCGGCGCATA TCCC(A)**T**$^{E\beta}$-5`

The 601 sequence is non-palindromic. To account for the observed asymmetry in nucleosome dynamics, we denote by α the left side of the sequence (forward strand) with base pairs counted in negative numbers from the fragment center. The other side is called β, with base pairs counted in positive numbers. Consistent with this nomenclature we refer to the (H2A-H2B) heterodimer attached to the α-side as D_α and the other heterodimer as D_β.

List of primers used for the DNA fragments

$I_\beta I_\alpha$

fwd 5`-CATGCACAGG ATGTATATAT CTGACACGTG CCT(Cy5)GGAGACT-3`

rev 3`-TAACT(A488)CGCC GGAGCCGTGG CCCTAAGAGG TCCCGCCGGC GCATATCCCA-5`

$E_\alpha Dy_\beta$

fwd 5`-T(A488)ATGCACAGG ATGTATATAT CTGACACGTG CCTGGAGACT AGGGAGTAAT CCCCT-3`

rev 3`-TCGCT(A594) ACGATCTCGA CAGATGCTGG TTAAC**T**CGCC GGAGCCGTGG CCCTAAGAGG TCCCGCCGGC GCATATCCCA -5`

$E_\beta Dy_\alpha$

fwd 5`-CATGCACAGG ATGTATATAT CTGACACGTG CCTGGAGACT AGGGAGTAAT CCCCTTGGCG GTTAAAACGC T(A594)GGGG -3`

rev 3`-CTCGA CAGATGCTGG TTAACTCGCC GGAGCCGTGG CCCTAAGAGG TCCCGCCGGC GCATATCCCT(A488)-5`

Dy_α

fwd 5`CATGCACAGG ATGTATATAT CTGACACGTG CCTGGAGACT AGGGAGTAAT CCCCTTGGCG GTTAAAACGC T(Cy5)GGGG- 3`

rev 3`-CTCGA CAGATGCTGG TTAACTCGCC GGAGCCGTGG CCCTAAGAGG TCCCGCCGGC GCATATCCCA-5`

Donor (A488) and acceptor (A594 or Cy5) dyes were attached to the Tymin bases marked in the primers via amino-C6 linkers

Analysis of multiparameter smFRET data

Intensity-based FRET efficiencies (E) were obtained by first correcting the measured green and red signal S_G and S_R for mean green and red background $\langle B_G\rangle$ and $\langle B_R\rangle$, spectral crosstalk (α = 5%), the ratio of green and red detection efficiencies (g_G/g_R = 0.8), donor and acceptor fluorescence quantum yields $\Phi_{FD(0)}$ = 0.80 and Φ_{FA} = 0.6, respectively. As detailed in [11], the final expression for the FRET efficiency based on corrected intensities is given by:

$$E = \frac{\frac{S_R - \alpha \cdot (S_G - \langle B_G \rangle) - \langle B_R \rangle}{g_R}}{\frac{\Phi_{FA}}{\Phi_{FD(0)}} \cdot \left(\frac{S_G - \langle B_G \rangle}{g_G} \right) + \frac{S_R - \alpha \cdot (S_G - \langle B_G \rangle) - \langle B_R \rangle}{g_R}} \quad \text{(Supplementary Equation 39)}$$

Computation of the theoretical static FRET line corrected for dye linker dynamics.

In the static case (i.e. molecules without dynamics) the FRET efficiency $E(\tau_{D(A)})$ can be described as a function of donor lifetime in the presence of acceptor dye:

$$E = 1 - \frac{\langle \tau_D \rangle_x}{\tau_{D0}}, \quad \text{(Supplementary Equation 40)}$$

where τ_{D0} is the donor fluorescence lifetime in the absence of acceptor and $\langle \tau_D \rangle_x$ is the mean species-averaged donor lifetime in the presence of acceptor. In the MFD analysis of single-molecule bursts, the maximum likelihood estimator determines only fluorescence-weighted average lifetimes $\langle \tau_D \rangle_F$ due to the low photon number (~100 photons per burst). To account for dye-linker dynamics, a numerical simulation was performed assuming Gaussian distribution of distances, $p(R_{DA})$ with mean and standard deviation $\bar{R}_{DA} \pm \sigma_{R_{DA}}$, to create an empirical relation between the species and fluorescence averaged lifetimes for the selected range of $\langle R_{DA} \rangle$ values (from 20 Å to 200 Å) using a 4th order polynomial function as described in [11]. Using this polynomial function the fluorescence-weighted average lifetimes $\langle \tau_D \rangle_F$ were converted to $\langle \tau_D \rangle_x$.. By substituting $\langle \tau_D \rangle_x$ in **Supplementary Equation 39** we can then represent the static FRET line as:

$$E = 1 - \frac{\sum_{i=0}^{4} C_i \left(\langle \tau_D \rangle_F \right)^i}{\tau_{D0}}, \quad \text{(Supplementary Equation 41)}$$

where C_i are the coefficients of the empirical polynomial function. Parameters for the static FRET line in main text Fig. 3b and 3c (orange lines) were: width of the linker broadening $\sigma_{R_{DA}} = 6\,\text{Å}$, τ_{D0} = 4.1 ns, $\Phi_{FD(0)}$=0.8, Φ_{FA}=0.32 and polynomial coefficients C_0 = -0.0211, C_1 = 0.434, C_2 = 0.3719, C_3 = -0.0798, C_4 = 0.0056 and $p\,(R_{DA}) = \frac{1}{\sqrt{2\pi} \cdot 2 \cdot \sigma_{R_{DA}}} \exp\left(\frac{-(R_{DA} - \bar{R}_{DA})^2}{2 \cdot \sigma_{R_{DA}}^{\ 2}} \right)$.

Computation of the dynamic FRET line corrected for dye linker dynamics.

For molecules with conformational dynamics the same procedure was applied to calculate a dynamic FRET line [11] where mixed fluorescent species were the result of an exchange between two fluorescent states with lifetimes $\langle \tau_1 \rangle_F$ and $\langle \tau_2 \rangle_F$:

$$E_{dyn} = 1 - \frac{\langle \tau_1 \rangle_F \cdot \langle \tau_2 \rangle_F}{\left(\langle \tau_1 \rangle_F + \langle \tau_2 \rangle_F - \sum_{i=0}^{3} C_i \left(\langle \tau_D \rangle_F \right)^i \right)} \quad \text{(Supplementary Equation 42)}$$

Parameters used in this work were:

Interconversion between MF* and HF (violet line): τ_{D0} = 4.1 ns, $\langle \tau_1 \rangle_F$ = 1.3 ns, $\langle \tau_2 \rangle_F$ = 2.6 ns, with polynomial coefficients C_0 = -1.3863, C_1 = 1.5072, C_2 = 0.0 and C_3 = 0.0.

Interconversion between MF* and MF (yellow line): τ_{D0} = 4.1 ns, $\langle \tau_1 \rangle_F$ = 2.6 ns, $\langle \tau_2 \rangle_F$ = 3.0 ns, with polynomial coefficients C_0 = -0.739, C_1 = 1.2252, C_2 =0.0 and C_3 = 0.0.

Supplementary References

1. Kalinin, S., Sisamakis, E., Magennis, S. W., Felekyan, S. & Seidel, C. A. M. On the origin of broadening of single-molecule FRET efficiency distributions beyond shot noise limits. *J. Phys. Chem. B* **114**, 6197-6206 (2010).
2. Felekyan, S., Kalinin, S., Sanabria, H., Valeri, A. & Seidel, C. A. M. Filtered FCS: species auto- and cross-correlation functions highlight binding and dynamics in biomolecules. *ChemPhysChem* **13**, 1036-1053 (2012).
3. Kalinin, S. *et al.* A toolkit and benchmark study for FRET-restrained high-precision structural modeling. *Nat. Meth.* **9**, 1218-1225 (2012).
4. Sindbert, S. *et al.* Accurate distance determination of nucleic acids via Förster resonance energy transfer: implications of dye linker length and rigidity. *J. Am. Chem. Soc.* **133**, 2463-2480 (2011).
5. Alberty, R. A. & Hammes, G. G. Application of the theory of diffusion-controlled reactions to enzyme kinetics. *J. Phys. Chem.* **62**, 154-159 (1958).
6. Eigen, M. & Hammes, G. G. Elementary steps in enzyme reactions (as studied by relaxation spectrometry). *Advances in Enzymology and Related Subjects of Biochemistry* **25**, 1-38 (1963).
7. Kim, J., Wei, S., Lee, J., Yue, H. & Lee, T. H. Single-molecule observation reveals spontaneous protein dynamics in the nucleosome. *J. Phys. Chem. B* **120**, 8925-8931 (2016).
8. Gansen, A. *et al.* Nucleosome disassembly intermediates characterized by single-molecule FRET. *Proc. Natl. Acad. Sci. U S A* **106**, 15308-15313 (2009).
9. Vasudevan, D., Chua, E. Y. & Davey, C. A. Crystal structures of nucleosome core particles containing the '601' strong positioning sequence. *J. Mol. Biol.* **403**, 1-10 (2010).
10. Gansen, A., Hieb, A. R., Böhm, V., Tóth, K. & Langowski, J. Closing the Gap between Single Molecule and Bulk FRET Analysis of Nucleosomes. *PLOS One* **8**, e57018 (2013).
11. Sisamakis, E., Valeri, A., Kalinin, S., Rothwell, P. J. & Seidel, C. A. M. Accurate single-molecule FRET studies using multiparameter fluorescence detection. *Methods Enzymol.* **475**, 455-514 (2010).
